# Genetic variation and heritability of grain protein deviation in European wheat genotypes

**DOI:** 10.1016/j.fcr.2020.107896

**Published:** 2020-09-15

**Authors:** Ellen F. Mosleth, Marie Lillehammer, Till K Pellny, Abigail J. Wood, Andrew B. Riche, Abrar Hussain, Simon Griffiths, Malcolm J. Hawkesford, Peter R. Shewry

**Affiliations:** a Nofima AS, P.O. Box 210, NO-1431 Ås, Norway; b Rothamsted Research, Harpenden, Hertfordshire AL5 2JQ, UK; c Department of Biosciences, COMSATS University Islamabad, Sahiwal Campus, Sahiwal, Punjab 57000, Pakistan; d John Innes Centre, Norwich Research Park, Colney Lane, Norwich NR4 7UH, UK

**Keywords:** Wheat, Nitrogen fertilisation, Grain protein deviation, Nitrogen use efficiency, Heritability

## Abstract

There is a well-established negative relationship between the yield and the concentration of protein in the mature wheat grain. However, some wheat genotypes consistently deviate from this relationship, a phenomenon known as Grain Protein Deviation (GPD). Positive GPD is therefore of considerable interest in relation to reducing the requirement for nitrogen fertilization for producing wheat for breadmaking. We have carried out two sets of field experiments on multiple sites in South East England. The first set comprised 11 field trials of 6 cultivars grown over three years (2008–2011) and the second comprised 9 field trials of 40 genotypes grown over two years (2015–2017) and 5 field trials of 30 genotypes grown in a single year (2017–2018). All trials comprised three replicate randomized plots of each genotype and nutrient regime. These studies showed strong genetic variation in GPD, which also differed in stability between genotypes, with cultivars bred in the UK generally having higher GPD and higher stability than those bred in other European countries. The heritability of GPD was estimated as 0.44, based on data from the field trials of 30 and 40 genotypes. The largest component contributing to the genetic variance was genotype (0.30), with a smaller contribution of the interaction between genotype and year/site (0.11) and a small (but statistically significant) contribution of nitrogen level. These studies suggest that selection for GPD is a viable target for breeders.

## Highlights

• Grain protein deviation has been determined for European wheat cultivars grown in multi-environment trials.
• Significant variation has been demonstrated, in mean GPD and in stability between environments.
• The variation in GPD has been apportioned between the effects of genotype, environment and G x E interactions.
• GPD is an attractive target for breeders to improve NUE and reduce the nitrogen requirement for breadmaking wheat.

## Graphical abstract

**Figure:**
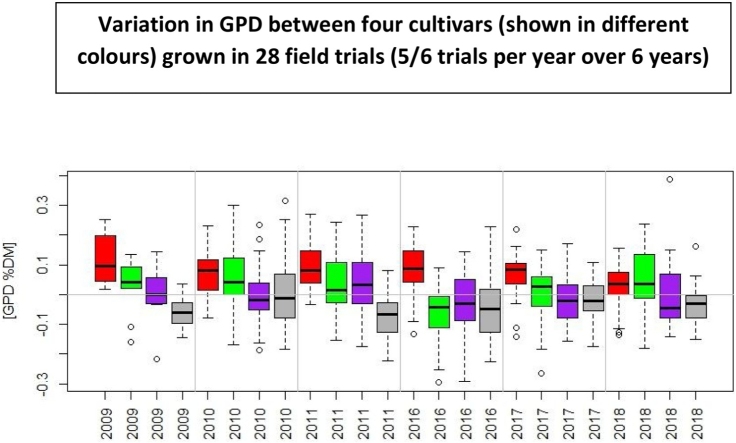

## 1 Introduction

Wheat is the most important food grain in temperate countries, accounting for about 20–50% of the total intake of calories in humans. Hence it plays a crucial role in food security and increases in production are required to feed the growing global population. With a fixed and limited landmass area available for wheat production the efficient utilisation of resources is critical. Nitrogen (N) is one of the major inputs for wheat production, being required to support the development of the crop canopy to maximise carbon capture and yield. However, it has been estimated that globally only 33 % of the applied nitrogen fertilization is recovered in the harvested grain (Raun and Johnson, 1999), which has led to a focus on nitrogen use efficiency (NUE). NUE is often defined as yield per unit available N, taking in account mineral N available in the soil and N applied as fertiliser. NUE is a product of the efficiency of N uptake (NUpE) multiplied by the utilisation efficiency (NUtE). The former is the entire above ground mass as a fraction of total N that is available, and the latter is the yield as a function of the N that is taken up (Moll et al., 1982; Hawkesford and Griffiths, 2019). Efficiency may be also considered as the recovery of N in the grain (Grain N), which is further influenced by the rate of remobilisation of N from vegetative tissue to the grain, and may be quantified as nitrogen harvest index (NHI). These traits have strong genetic components, with a study of 56 breeding lines and cultivars from the eastern US wheat region giving heritabilities of 0.64 for NUE, 0.58 for NUtE, 0.29 for NUpE, and 0.29 for NHI (Hitz et al., 2017).

Nitrogen fertilization is particularly important for wheat production when grain is used for breadmaking as total protein content is important in addition to protein quality (Shewry, 2007). Consequently, the amount of nitrogen required for growing breadmaking wheat may be above the optimum required for grain yield. This is clearly undesirable in terms of cost and sustainability, and there is considerable interest in reducing the nitrogen requirement for producing bread-making wheat. Although it may be possible to modify the processing conditions to allow the use of grain with a lower protein content for breadmaking, a minimum protein concentration is required, and high protein content can to some extent compensate for adverse effects of the environment on protein quality.

In general, there is a well-established negative relationship between the yield and the concentration of protein in the grain which can be explained by competition for carbon and nitrogen in the grain, and by dilution of protein by starch in modern high yielding genotypes. However, some wheat genotypes consistently deviate from this relationship, a phenomenon known as Grain Protein Deviation (GPD) (Monaghan et al., 2001). GPD is defined as the deviation from the negative regression between grain protein and yield as described by Monaghan et al. (2001). GPD is negative in wheats developed to have high contents of starch and low contents of protein for distilling or livestock feed, and positive in some high protein wheat genotypes bred for breadmaking.

GPD is known to be under genetic control with variation between genotypes (Bogard et al., 2010; Latshaw et al., 2016; Oury and Godin, 2007), and several QTLs associated with increased GPD have been identified by genome-wide association studies (Nigro et al., 2019). However, the relationship between yield and grain % N may be masked by environmental effects, as shown in a study of multi-site trials over 14 years (consisting of 82 site x year combinations) (Oury et al., 2003). Although variation was observed between the growing sites, a strong negative relationship was revealed between grain yield and grain %N across these site/year combinations when groups of genotypes with high genetic variability for the two traits were considered.

Some studies have shown genetic variation in GPD is related to differences in post-anthesis N uptake (Monaghan et al., 2001; Bogard et al., 2010). Bogard and co-workers (Bogard et al., 2010) performed experiments over 27 different growth environments in northern France, sampling wheat at anthesis and at harvest to calculate post-anthesis N uptake, N remobilization, and N remobilization efficiency. They found that the relationship between GPD and post-anthesis N uptake varied between the different growth conditions independently of anthesis date, with GPD and anthesis date being significantly correlated in only 5 of the 27 growth environments. On the other hand, the post-anthesis N uptake rather than remobilization or total N content at anthesis was significantly positively correlated with GPD in 12 of the growth environments, with correlation coefficients ranging from 0.44 to 0.76, and close to significant in others. Mosleth et al. (2015) compared the patterns of gene expression in developing grain of six genotypes differing in GPD, identifying gene transcripts which were associated with both positive and negative GPD.

The analysis of GPD is challenging because both grain protein and grain yield exhibit strong genotype x environment (G x E) interactions, including effects of N fertilization where this is included as a factor. We therefore developed a statistical approach to dissociate differences in grain protein content from direct effects of nitrogen availability and indirect effects of yield (Mosleth et al., 2015). This analysis was initially applied to a set of 6 cultivars grown on two sites over three growth years. We now use the same approach to determine GPD in a much larger dataset, comprising 40 genotypes and 10 sites, allowing us to partition the variance in GPD between genotype, environment and G x E (E being both N application and location) to quantify the heritability and impact of the genotype on GPD. We also include unpublished data from additional sites from our previous study (Mosleth et al., 2015) for comparative purposes.

## 2 Materials and methods

### 2.1 Wheat lines

The present study comprised two sets of wheat genotypes, each grown on several sites over a period of three years.

The first set comprised the six cultivars Cordiale, Hereward, Istabraq, Malacca, Marksman and Xi19. All are winter wheats, with Istabraq being a feed wheat and the others breadmaking wheats (Mosleth et al., 2015; Chope et al., 2014).

The second set comprised 40 wheat genotypes selected to represent a range of types, with a focus on winter breadmaking wheats (Supplementary Material Table S1). These included 9 current UK breadmaking wheats (nabim groups 1 and 2), 2 UK feed wheats (nabim group 4), 4 UK spring wheats, 8 older UK breadmaking wheats, 2 French hybrid wheats, 2 high protein cultivars from Hungary, 10 cultivars from other EU countries (France, Germany and Denmark) and three mutant lines of the UK spring breadmaking wheat Paragon: 1BL/1RS, Stay Green and RhtD1b. These three mutant lines were included because all would be expected to have positive effects on grain yield, which could in turn affect grain nitrogen content (Flintham et al., 1997; Villareal et al., 1995; Kipp et al., 2014). It is therefore of interest to determine whether they also differ in GPD.

### 2.2 Field trials

Two sets of field trials were carried out, with different sets of cultivars and levels of nitrogen application.

The first set was a total of 11 growth environment, comprising three seasons (2008−9, 2009−10, 2010−11) and five sites in the UK: (Harpenden, Hertfordshire, 51° 48′ 19.79″ N 0° 21′ 11.39″ E), KWS (Thriplow, Cambridgeshire, 52° 5′ 49.866′' N, 0° 6′ 18.7452′' E), Limagrain (Woolpit, Bury St Edmunds, Suffolk, 52° 13′ 39.18′' N, 0° 52′ 45.03′' E), RAGT (Ickleton, Cambridgeshire, 52° 3′ 39.8952′' N, 0° 8′ 42.8604′' E), and Syngenta (Duxford, Cambridgeshire, 52° 11′ 9.0456′' N, 0° 11′ 39.3396′' E). Three replicate plots were grown at three nitrogen levels: 100 kg/Ha, 200 kg/Ha and 350 kg/Ha. Further experimental details and analyses of the grain samples to determine GPD and gene expression profiles have been described previously (Mosleth et al., 2015).

The second set was grown over three seasons (2015−16, 2016−7, 2017−18) at six sites in the UK: Rothamsted Research (Harpenden, Hertfordshire, 51° 48′ 19.79″ N 0° 21′ 11.39″ E), Agrii (Throw’s Farm, Essex, 52° 10′ 19.2′' N, 0° 17′ 2.4′' E, 52° 10′ 55.2′' N, 0° 17′ 2.4′' E and 52° 11′ 13.2′' N, 0° 15′ 39.6′' E in 2015−6, 2016−7 2017−8, respectively), Limagrain (Woolpit, Suffolk, 52° 13′ 39.18′' N, 0° 52′ 45.03′' E), KWS (Thriplow, Hertfordshire, 52° 5′ 49.866′' N, 0° 6′ 18.7452′' E), Saaten Union (Newmarket, Suffolk, 52° 9′ 39.6′' N 0° 27′ 39.6′' E) and DSV (Wardington, Oxfordshire, 52° 06′ 39.9″ N 1° 18′ 23.6″ W, 52° 06′ 45.6″ N 1° 17′ 17.3″ W and 52° 06′ 20.0″ N 1° 20′ 35.5″ W in 2015−6, 2016−7 2017−8, respectively. All 40 genotypes were grown at a total of 9 sites over 2 years (2015−6 and 2016−7) and a sub-set of 30 genotypes on 5 sites for a third year (2017–2018).

All lines (spring and winter type) were planted in October and each trial comprised three randomised replicated plots of 6 × 1.5 m at each nitrogen level with a seed rate of 250/m^2^. Soil residual nitrogen varied between sites and years with means of 46, 72 and 65 kg/Ha in 2015−16, 2016−7, 2017−18, respectively. Two levels of nitrogen fertilisation (150 and 250 kgN/ha) were applied as splits (50 + 50 + 50 kg/Ha or 50 + 150 + 50 kg/Ha, respectively) to separate blocks with all plots also receiving 40 kg S/ha.

Other agronomic treatments were standard for the sites. Material from the KWS trials in 2017 and 2018 and the DSV trial in 2017 were discarded due to technical problems. There were 14 year-sites in total. The yields were converted to tonnes/Ha and the N contents of the grain samples determined by NIR. Grain protein was calculated as N x 5.7.

### 2.3 Statistical methods

GPD was calculated for each level of fertiliser application as deviation from the well-known negative relation between grain %N and yield after adjusting both grain %N and yield for the impact of N fertilization as described by Mosleth et al. (2015). Correction of %N for applied N fertiliser gave grain%N_corrN while correction of yield gave yield_corrN. A linear model between grain%N_corrN and yield_corrN was then used to calculate GPD, which is the residual of the linear model between Grain%N_corrN and Yield_corrN. The analysis was performed within each site to allow investigation of how GPD varies across different environmental conditions.

In mathematical terms our approach for calculation of GPD is a one-step procedure when applied to one N level at the time and as a three-step procedure when applied to more than one N-level

Calculation of GPD using data for a single N level:

*Step 1. Let y be grainN, x_0_ be intercept and x_1_ be the yield*

y = x_0_ + b*x_1_

The residual of this model is GPD.withinN

Calculation of GPD using data for more than one N level by first adjusting for the effect of N:

*Step 1. Let y be grain%, x_0_ be intercept and x be the N level*

y = x_0_ + b*x_1_

*The residual of this model is grain%N_corrected for N (grai%N_corrN)*

Step 2. Let y be yield, x_0_ be intercept and x_1_ be the N level

y = x_0_ + b*x_1_

*The residual of this model is yield_corrected for N (yield_corrN)*

*Step 3. Let y be grain_corrN, x_0_ be intercept and x_1_ be the yield_corrN*

y = x_0_ + b*x_1_

The residual of this model is GPD

For estimation of the heritability and the genetic variance components of GPD, the data were analysed in ASReml (VSN, Hemel Hempstead, UK). In these calculations, the cultivars are considered as random selections of a population of wheat cultivars.

The analysis was both performed within each site according to the mixed model:

(Model 1) y=μ+b*N+CV+CV*N+e

and across all sites using the following univariate linear mixed model:

(Model 2) y=μ+ys+b*N+ys*N+CV+CV*ys+CV*N+CV*N*ys+e

where *y* is the recorded phenotype (Yield_corr, grain_corr or GPD), μ is the general mean, y*s* is the effect of a combined factor for years and sites “year_site”, fitted as a fixed effect, *N* is the level of N-fertilization and b the estimated regression coefficient of N on the phenotype, *CV* is the genotype, fitted as an independent normally distributed random effect with mean 0 and variance $\sigma_{add}^{2}$. CV*ys and CV*N are the interaction effects of genotype by year_site and genotype by N-fertilization, CV*N*ys is the three-way interaction effect between genotype, year_site and N-fertilization and *e* is the random residuals ∼N(0,$\sigma_{e}^{2}$). The interaction terms were also assumed to be independent and normally distributed with means 0 and variances $\sigma_{CV\_ys}^{2}$ for the interaction between CV and year_site, $\sigma_{CV*N}^{2}$ for the interaction between CV and N-level and $\sigma_{CV*N*ys}^{2}$ for the three-way interaction.

Across sites, additive heritability was estimated from Model 2 as:

$$\frac{\sigma_{add}^{2}}{\sigma_{add}^{2}+\sigma_{CV*ys}^{2}+\sigma_{CV*N}^{2}+\sigma_{CV*N*ys}^{2}+\sigma_{e}^{2}}$$

This heritability reflects the component of the genetic variance that are generic and independent of year_sites.

To estimate the fraction of total variation that resulted from genotype by environment interactions, the heritability of the interaction terms were estimated as

$$\frac{\sigma_{CV*ys}^{2}}{\sigma_{add}^{2}+\sigma_{CV*ys}^{2}+\sigma_{CV*N}^{2}+\sigma_{CV*N*ys}^{2}+\sigma_{e}^{2}}$$

for the interaction with year_site and as

$$\frac{\sigma_{CV*N}^{2}}{\sigma_{add}^{2}+\sigma_{CV*ys}^{2}+\sigma_{CV*N}^{2}+\sigma_{CV*N*ys}^{2}+\sigma_{e}^{2}}$$

for the interaction with N-level, and

$$\frac{\sigma_{CV*N*ys}^{2}}{\sigma_{add}^{2}+\sigma_{CV*ys}^{2}+\sigma_{CV*N}^{2}+\sigma_{CV*N*ys}^{2}+\sigma_{e}^{2}}$$

for the three-way interaction.

We estimate the total heritability (i.e. the fraction of phenotypic variation described by genetics, including interactions terms) as

$$\frac{\sigma_{add}^{2}+\sigma_{CV*ys}^{2}+\sigma_{CV*N}^{2}+\sigma_{CV*N*ys}^{2}}{\sigma_{add}^{2}+\sigma_{CV\_ys}^{2}+\sigma_{CV\_N}^{2}+\sigma_{CV*N*ys}^{2}+\sigma_{e}^{2}}$$

Within sites, variance components

$$\sigma_{add}^{2}$$

and

$$\sigma_{CV\_N}^{2}$$

were derived for all sites based on estimates from Model 1.

Both variation in the heritability of GPD for cultivars as calculated within each site and the significance of the interaction term between CV and year/site as calculated across sites will reflect variation in the genetic variance components of GPD and hence reflect changes in the ranking of cultivars across different sites.

We also applied ANOVA for testing the four cultivars that were included in both data sets (the year of growth 2009–2011 and 2016–2018, respectively). In these analyses, the cultivars are fixed effects in the ANOVA.

## 3 Results

### 3.1 Determination of GPD in field trials

**Fig. 1:**
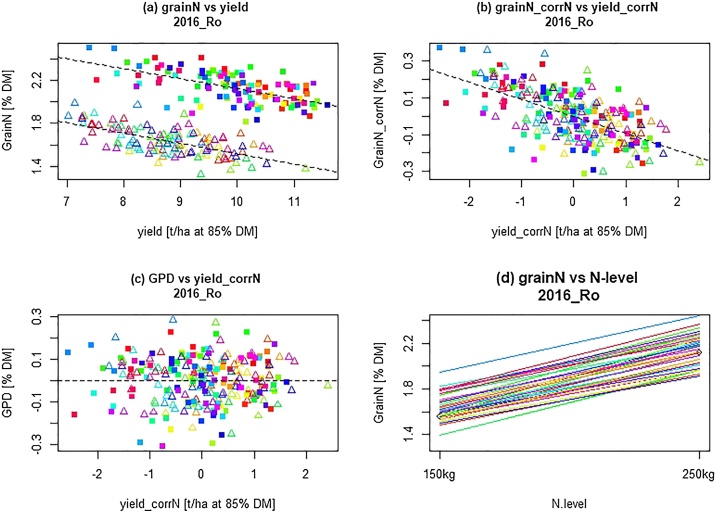
Plots for 40 genotypes grown at Rothamsted in 2015-16, showing (a) grain %N vs yield, (b) grain %N corrected for nitrogen and yield vs yield, (c) GPD vs yield corrected for impact of N-levels and (d) grain N vs N level. The 40 cultivars (rainbow color-coded) were grown at two N-levels: 150 kg/ha (open triangles), and 250 kg/ha (filled squares).

Grain N was initially plotted against yield for each site and environment, as illustrated for the 40 genotypes grown at Rothamsted in 2015−16 (Fig. 1a). This showed a clear negative correlation between yield and grain %N, with two parallel clusters of data points corresponding to the samples grown at high (250 kgN/Ha) and low (150 kgN/Ha) levels of N fertiliser level. When calculating GPD within the site, there were no significant interactions between cultivar and N-level within the sites (Fig. 1d). For the rest of this publication, we therefore used the GPD that was adjusted for the effects of N within each site.

**Fig. 2:**
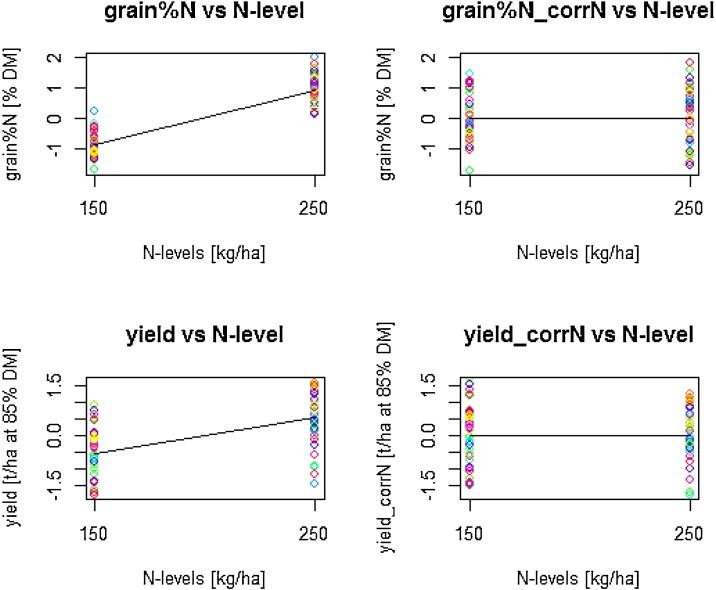
Correction of grain %N and yield for the impact of the level of N-fertilization shown for 40 wheat cultivars grown at Rothamsted Research in 2015-2016. The upper panels show grain %N before (left) and after (right) the correction. The lower panels show yield before (left) and after (right) the correction.

The positive effects of N level on grain%N and on yield were corrected within each site as described previously (Mosleth et al., 2015) and illustrated for the 40 cultivars grown at Rothamsted Research in 2015−16 (Fig. 2). The upper panels show grain %N before and after the correction and the lower panels yield before and after the correction.

When the corrected values for grain %N and yield were plotted, the negative relationship between the two traits was retained but the difference between the fertiliser levels was abolished with the data points forming a single cluster (Fig. 1b). This allowed the calculation of GPD as the residual from the negative regression of grain%N_corrN on yield_corrN, as displayed in Fig. 1c.

Similar plots are made for each experimental site and year of growth for both the small set of 6 genotypes and the large set of 40 genotypes (Supplementary Figures S1 and S2, respectively). As the number of genotypes are different in the two data sets, GPD cannot be directly compared across the data sets, and heritability is only provided for the data set with the largest number of cultivars. As expected by definition, GPD was positively related to grain %N (p (corr) < 0.001) in all datasets, with no relationship to the yield_corrN (p(corr) > 0.001). The variance components of the input factors cultivar (V.cv) and the interaction between cultivar and N-level (V.n.cv) for each data set are displayed as pie diagrams together with the residual variance (V.res) in Supplementary Figures S1 and S2.

**Fig. 3:**
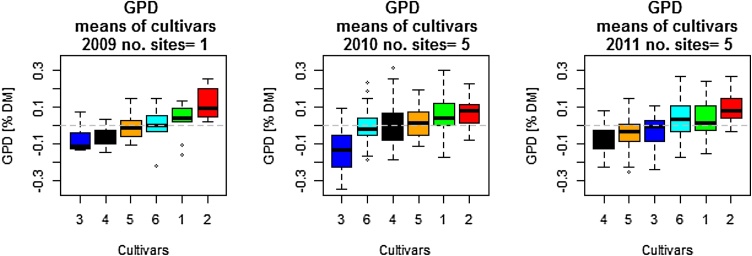
Box plot of GPD for the set of six cultivars for each year of growth (2008-2009, 2009-2010 and 2010-2011) as means across growth sites within each year, N fertilizing level and rep for the six cultivars: 1, Cordiale; 2, Hereward; 3, Istabraq; 4, Malacca; 5, Marksman; 6, Xi19 ranked by their median. The boxes are the interquartile range (IQR) 25 % percentile to the 75 % percentile, covering 50 % of the values within the boxes, and the whiskers are marked outside 1.5 times the IQR.

**Fig. 4:**
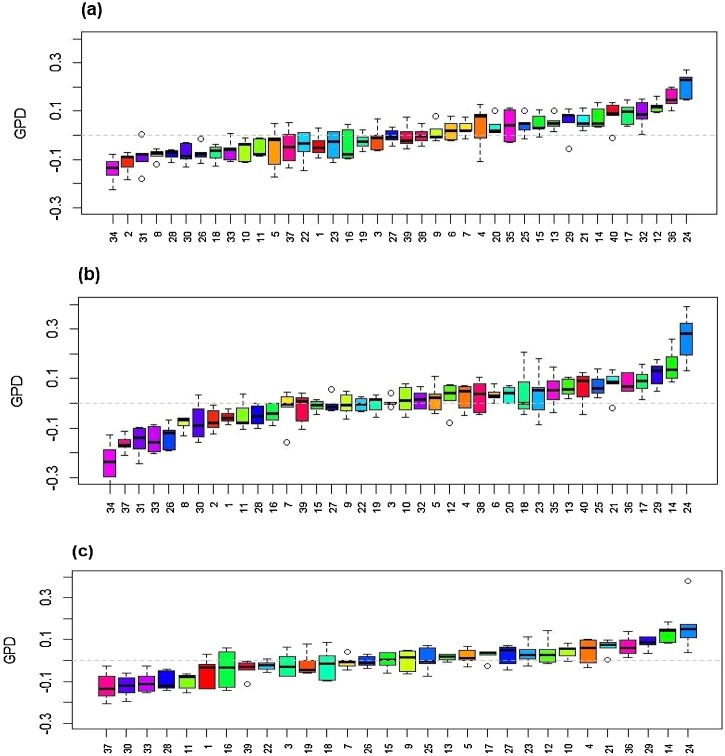
Boxplot of GPD for the sets of 40 and 30 genotypes as means of each site and year for (a) 2015-2016, (b) 2016-2017 and (c) 2017-2018. Cultivars are numbered as in Supplementary Material Table S1: 1, JB Diego; 2, Dickens; 3, Skyfall; 4, Crusoe; 5, Gallant; 6, Solstice; 7, KWS Trinity; 8, Einstein; 9, KWS Cashel; 10, Cordiale; 11, KWS Lili; 12, Mulika; 13, Paragon; 14, Granary; 15, KWS Willow; 16, KWS Siskin; 17, Hereward; 18, Soissons; 19, Xi19; 20, Avalon; 21, Cadenza; 22, Malacca; 23, Shamrock; 24, Mv Karisma; 25, Mv Lucilla; 26, Memory; 27, Potenzial; 28, Rumor; 29, Nelson; 30, Hybery SU; 31, Hystar; 32, Tobak; 33, Apache; 34, Arlequin; 35, Premio; 36, Genius; 37, Dacanto; 38, Paragon 1BL/1RS; 39, Paragon Stay Green; 40, Paragon RhtD1b.

Box plots of GPD for the six cultivars grown in 2009, 2010 and 2011 are shown in Fig. 3, and for the 40 genotypes grown in 2016 and 2017 and the 30 grown in 2018 in Fig. 4. There is consistent variation in GPD across the different years and sites. For example, Hereward, which is included in all sets of material, exhibits high GPD in all sites and year of growth. Other genotypes in the set of 40 which exhibit high positive GDP are Mv Karisma (high protein type from Hungary), Granary (UK), Genius (Danish) and Nelson (German) in all three years of growth, while Arlequin (France), Hystar (French hybrid), Dacanto (Denmark) and Apache (France) show strong negative GPD.

**Fig. 5:**
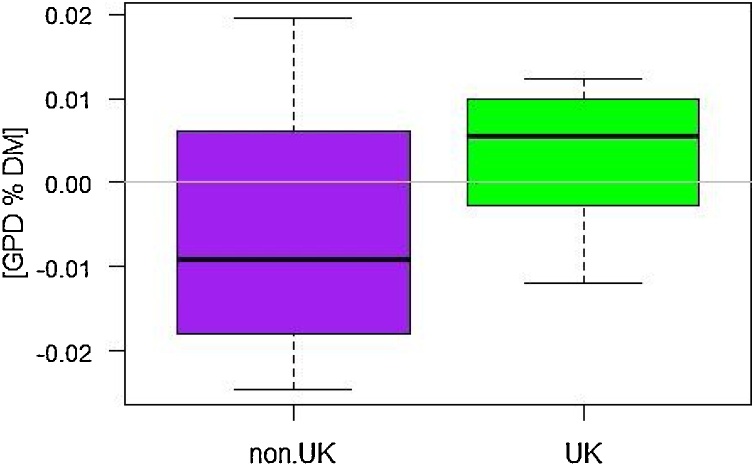
Box plot comparing GPD of cultivars bred outside the UK (non.UK) and in the UK. The variation within the groups reflect variation across different year_sites as means of N-level and replicates within each year_site.

Comparison of the 14 cultivars bred outside the UK with the 26 UK lines showed that the mean GPD of the latter was higher (as shown by the box plot in Fig. 5), which is consistent with their better adaptation to UK conditions.

The stay green mutant of Paragon showed little or no GPD, compared to the positive GPD displayed by the parental Paragon line, which was expected because stay green lines have an extended photosynthetic period and a reduced period of senescence.

The lines also differed in their ranges of GPD, indicating differences in stability of the trait. Five lines showed particularly high stability: Einstein (UK group 2), Skyfall (UK group 1), Solstice (UK group 1) and Paragon (UK group 1 spring type). The non-UK bred lines showed wider ranges for GPD (Fig.5), with Decanto (Denmark) Mv Karizma (Hungary) and Arlequin (France) being the most variable. This again may reflect the fact that they are less well adapted to the UK growth conditions.

Of the 40 lines studied here, only four were included in the two sets of material: Hereward, Consort, Malacca, and Xi19. It is therefore of interest to compare the performance of these four cultivars in the two studies. Tukeys test of pairwise comparisons across all sites (p < 0.05) using one way-ANOVA with cultivars as fixed effects showed that Hereward had significantly higher GPD than the three other cultivars, and Malacca lower GPD than the three other cultivars, whereas Cordiale and Xi19 are not significantly different from each other (Supplementary Table S2).

**Fig. 6:**
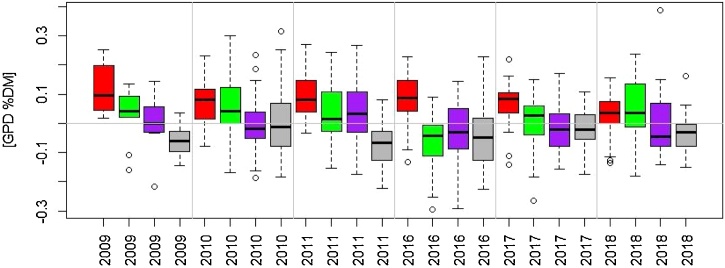
Box plots of GPD for the four cultivars grown in all six year: Hereward (red), Cordiale (green), Xi19 (purple), and Malacca (grey). Each box represent variation across the sites and replicates within each year and cultivar. (For interpretation of the references to colour in this figure legend, the reader is referred to the web version of this article).

Although genotype*environmental interactions were observed, Hereward had positive GPD in all years when considering each year separately (Fig. 6), and also when considering each year_site (Suppl Fig. S3). The median of GPD for Hereward was below zero for only one out of 13 year_sites.

### 3.2 Heritability of GPD

**Fig. 7:**
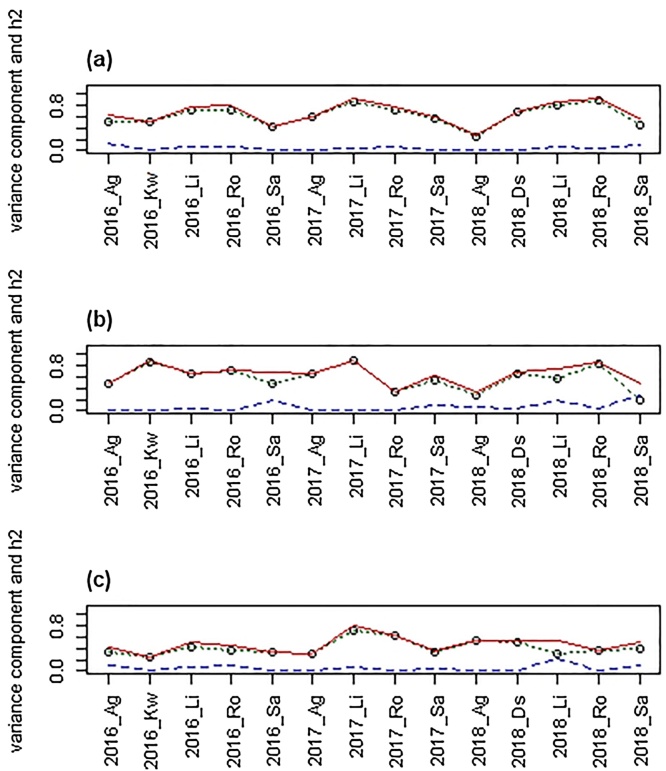
Variance components of (a) grain_corrN, (b) yield_corrN and (c) GPD, displaying the main effects of the component of the cultivars (green, dotted lines), the interaction between cultivar and N-fertilization (blue, hatched lines) and the heritability (red, solid line). Years are 2016, 2017and 2018; sites are Ag, Agii; Kw, KWS; Li, Limagrain; Sa, Saaten Union; Ro, Rothamsted; DS, DSV. (For interpretation of the references to colour in this figure legend, the reader is referred to the web version of this article).

The heritability of GPD and the genetic variance components were estimated for the second set of genotypes, both within each site and across all sites, using a univariate linear mixed model (Table 1). The variance components calculated within each year_site for grain_corrN, yield_corrN and GPD (Fig. 7) shows that they vary across the year_sites

**Table 1:** Estimated variance components for genotype (G), the interaction between G and N fertilizer level (N), the interaction between G and year_site (YS), and the three-way interaction as fraction of total phenotypic variance for dataset 2 (40 cultivars).

Trait	G	G x N	G x YS	Gx YSx N	Sum
**Yield_corr**	0.42***	0.002^NS^	0.21***	0.06***	0.69
**grainN_corr**	0.48***	0.025***	0.17***	0.01^NS^	0.69
**GPD**	0.30***	0.030**	0.11***	0.00^NS^	0.44

*** p-value less than 0.001; ** is p-value less than 0.01, * is p-value less than 0.05. Fixed effects of YS, N and the interaction term YS x N were fitted.

Each variance component gives the fraction of the total phenotypic variance explained by each random effect in the model. The sum gives the total heritability of the trait. This showed that the total heritability of GPD in this study was 0.44, i.e. nearly half of the observed variation in GPD was due to genetic differences between the cultivars. The largest genetic component is the main effect of genotype (0.30), which is the part of the genotypic effect that is not affected by nitrogen level or year-site. In addition, a proportion of the genetic variance was due to the interaction effect between genotype and year/site (0.11). A small, but significant, proportion of the variance was explained by interaction between genotype and nitrogen level, but no three-way interactions between genotype, year-site and nitrogen level were found (i.e. the genotypes seem to react similarly to changes in nitrogen across year sites).

Eleven out of the 17 individual field trials gave higher estimates for heritability than that estimated for the combined dataset, with the heritability between the trials varying from 0.25 to 0.84. The heritability of GPD was lower than the heritabilities of yield and grain N, which were both 0.69.

## 4 Discussion

Grain protein deviation is of great interest to breeders, agronomists, farmers and grain processors as it provides a clear opportunity to improve the utilisation of nitrogen by breadmaking wheats, hence reducing the requirement for nitrogen application with associated impacts on the cost of production and environmental footprint.

The analyses of genotypes grown in multi-environment (site and year) trials show that there is strong genetic variation in GPD, implying that it should be amenable to selection by wheat breeders (Monaghan et al., 2001; Bogard et al., 2010; Mosleth et al., 2015). It is notable that there were no significant interactions between genotype and N fertiliser level within each site, with the ranking of the cultivars being similar at the different levels of N fertiliser level that were applied (ranging from 100−350 kgN/ha). However, there were significant interactions between genotype and the years and sites, which means that the ranking of the cultivars was affected by the growth environment. Cultivars with stable high GPD across the growth environments were nevertheless observed. This is consistent with the heritability, which was calculated as 0.44 for the set of 30/40 cultivars.

However, it should also be noted that the heritability calculated for a sample set is a property of the populations and the environment. The cultivars in this study were selected to represent variation in processing quality at low nitrogen applications, with some being bred in the UK, and hence well adapted to the growth conditions, and others being bred in other European countries. By contrast, the sites and years (environments) should represent the range of conditions usually encountered in the major wheat-growing area in the south-eastern part of the UK. The genotypes and environments are not, therefore, representative of global variation, and the heritability estimate is not a general estimate for GPD in wheat. Nevertheless, it is a more realistic estimate than the heritability estimated from a single trial, where G by E interactions will not be considered and the random variation is much smaller (and hence the heritability higher). It should also be noted that the UK cultivars displayed less variation in GPD across the different sites than those bred in other countries.

About a quarter of the genetic variation in the 14 field trials was related to interactions between genotype and trial, meaning that cultivars differed in ranking between trials. By contrast, although the interaction between genotype and fertilization level was statistically significant, as calculated by the mixed model across all sites, the magnitude was small. Despite these interactions, about three quarter of the total genetic variance was ascribed to the cultivar itself, suggesting that selection for GPD by wheat breeders is a realistic target, despite some variation in relative performance between years. The ability to develop cultivars with consistently high GPD is illustrated by Hereward, which showed high and stable GPD in both sets of samples.

Two recent studies in durum wheat indicate the challenges in exploiting the genetic variation and heritability of GPD to develop improved cultivars. Rapp et al. (2017) carried out genome wide association mapping of two panels (comprising 159 and 189 genotypes) grown in multiple locations. They showed complex genetic architecture with most QTLs having small effects and being detected in only one panel. GPD showed strong positive correlation with protein content, indicating that the main impact of direct selection for GPD would be increased total protein content. Nigro et al. (2019) studied a panel of 240 genotypes grown in 7 field trials. Eleven QTLs for grain protein content were stable in at least three environments, of which four were also associated with positive GPD. Hence, in practical terms it should be possible to carry out a preliminary selection for high grain protein content, followed by second selection for GPD.

In conclusion, we have shown that wide variation in GPD exists between cultivars, and that the differences are largely reproducible between harvest years. About 44 % of this variation is heritable and should therefore be amenable to exploitation by breeders to develop improved cultivars.

## CRediT authorship contribution statement

**Ellen F. Mosleth:** Conceptualization, Methodology, Formal analysis, Writing - original draft. **Marie Lillehammer:** Methodology, Formal analysis, Writing - review & editing. **Till K Pellny:** Supervision, Investigation. **Abigail J. Wood:** Investigation, Data curation. **Andrew B. Riche:** Investigation, Supervision. **Abrar Hussain:** Investigation. **Simon Griffiths:** Conceptialization, Writing - review & editing. **Malcolm J. Hawkesford:** Conceptialization, Supervision, Writing - review & editing. **Peter R. Shewry:** Conceptionalisation, Supervision, Project administration, Writing - original draft, Writing - review & editing.

## Declaration of Competing Interest

The authors declare that they have no known competing financial interests or personal relationships that could have appeared to influence the work reported in this paper.
