# Supplementary material for: Genetic variation and heritability of grain protein deviation in European wheat genotypes
Source: Field Crops Res. 2020 Sep 15;255:107896. doi: 10.1016/j.fcr.2020.107896 (PMC7397848; doi:10.1016/j.fcr.2020.107896)
Supplement: Supplementary file 2 [file mmc2.docx]

$\frac{{}}{{}{}{}{}{}}$ $\frac{{}}{{}{}{}{}{}}$**Appendix A. Supplementary data**

**Table S3**. Full datasets (samples, sites, years, grain nitrogen yield, corrected nitrogen and GPD) for genotypes grown in 2015-2016, 2016-2017 and 2017-2018.

**Fig. S1.** Analysis of data for the six cultivars grown on the individual sites in 2008-2009, 2009-2010 and 2010-2011.

The scatter plots (top) show (from left to right) Grain%N vs yield, grain%N corrected for N-fertilization vs grain yield corrected for N-fertilization, GPD vs yield corrected for N-fertilization and GPD vs grain%N corrected for N-fertilization.

The pie charts show the relative proportion of the variance within each year accounted for by cultivar (V.cv), the interaction between cultivars and grain%N and the residual within each site within each year.

**Fig. S2.** Analysis of data for the 40 genotypes grown on the individual sites in 2015-16 and 2016-2017 and for the 30 genotypes grown on the individual sites in 2017-2018.

The scatter plots (top) show (from left to right) Grain%N vs yield, grain%N corrected for N-fertilization vs grain yield corrected for N-fertilization, GPD vs yield corrected for N-fertilization and GPD vs grain%N corrected for N-fertilization.

The pie charts (bottom) show the relative proportion of the variance within each year accounted for by cultivar (V.cv), the interaction between cultivars and grain%N and the residual within each site within each year.
