# Supplementary figures and images for: Genetic variation and heritability of grain protein deviation in European wheat genotypes
Source: Field Crops Res. 2020 Sep 15;255:107896. doi: 10.1016/j.fcr.2020.107896 (PMC7397848; doi:10.1016/j.fcr.2020.107896)

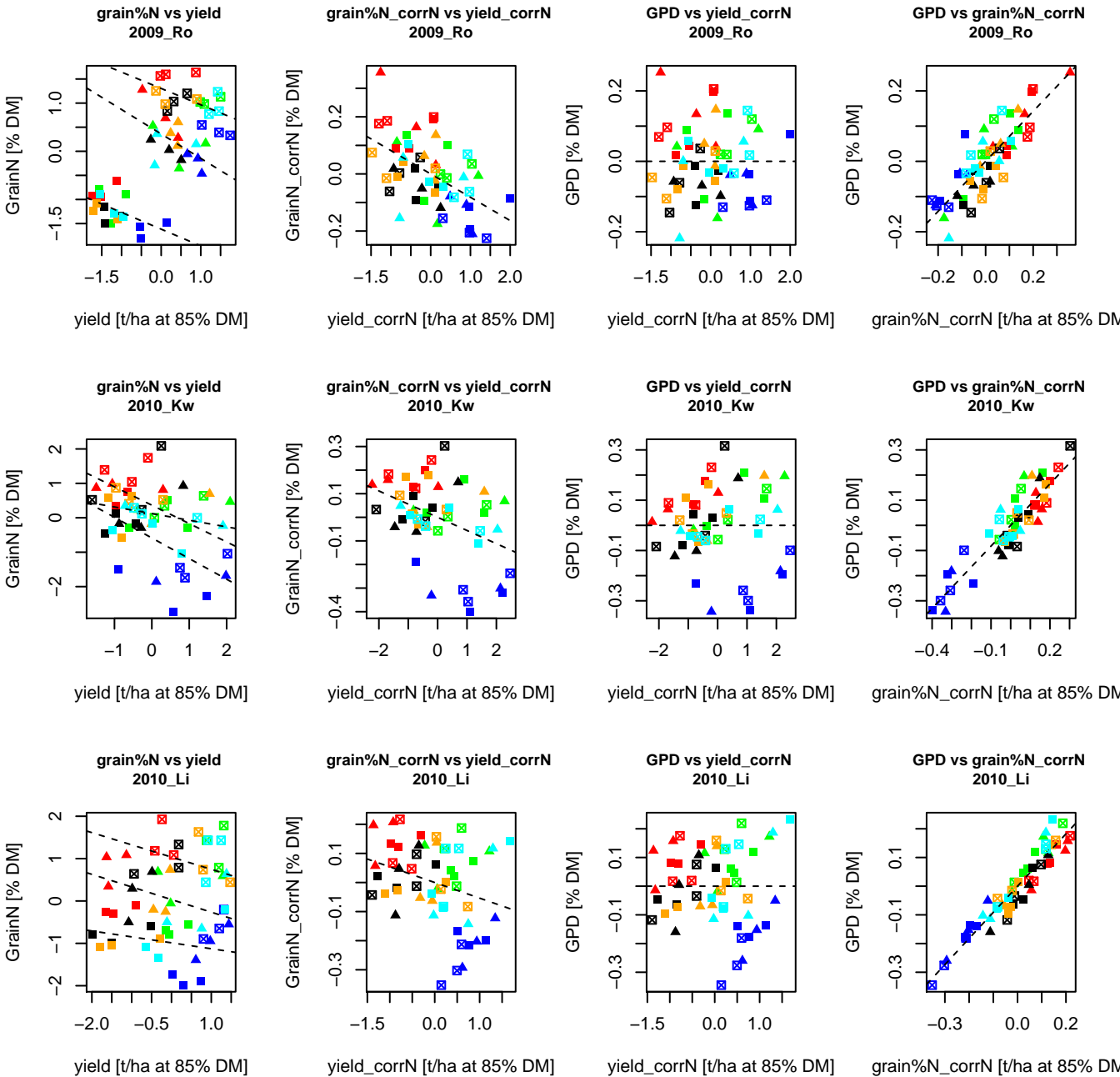

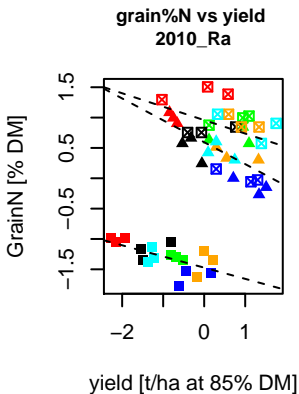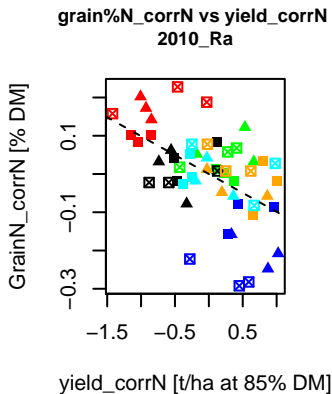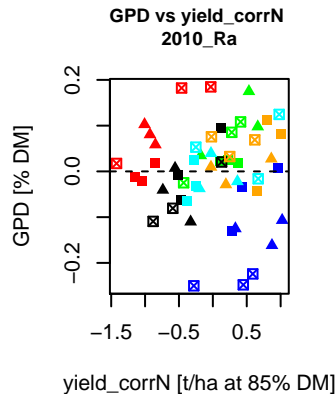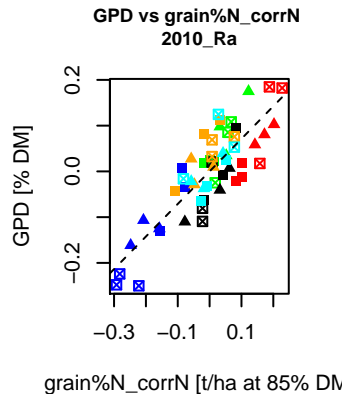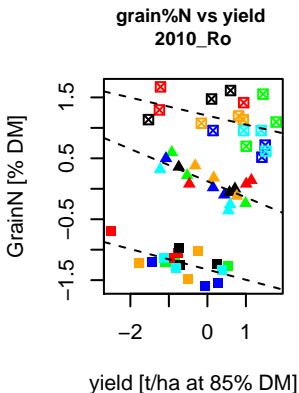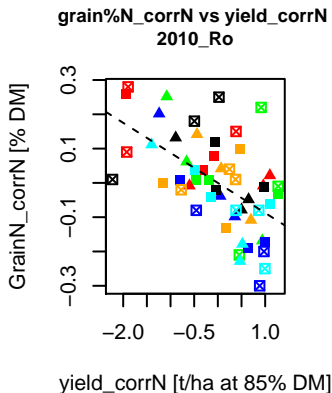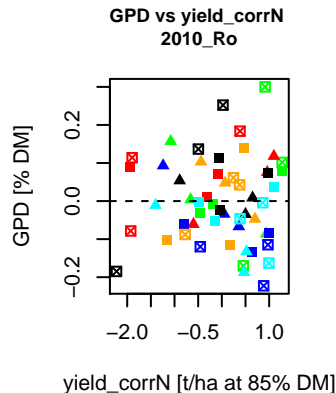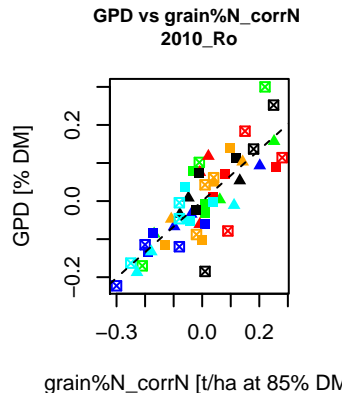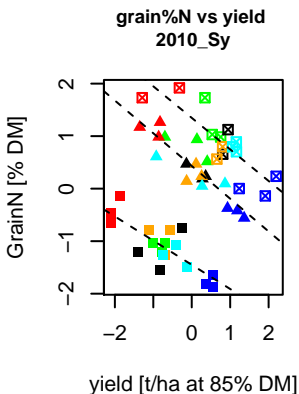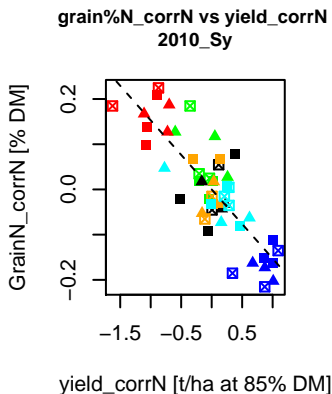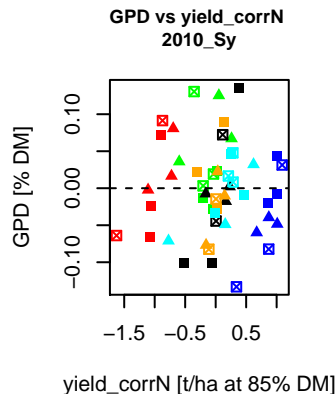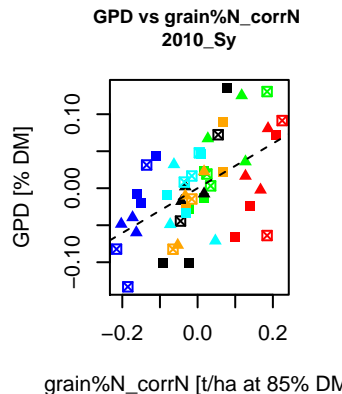

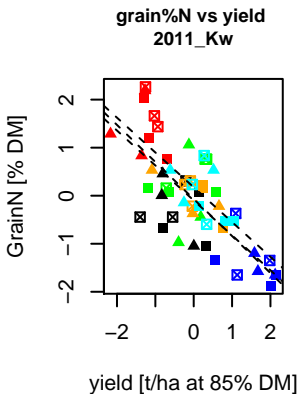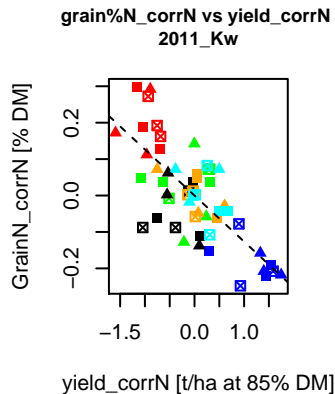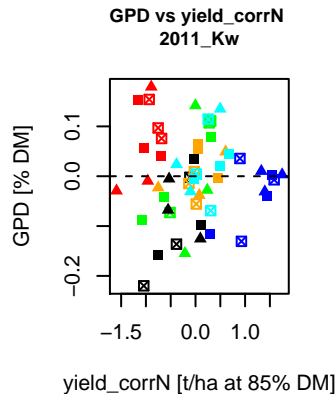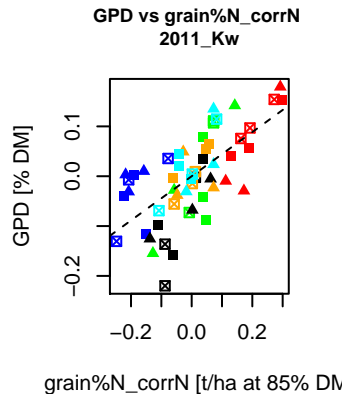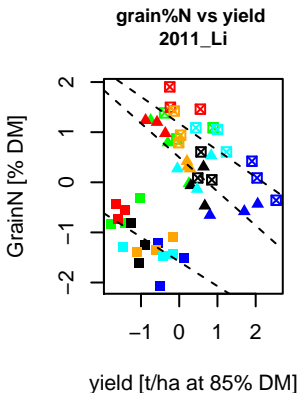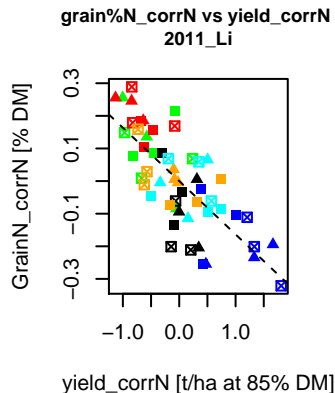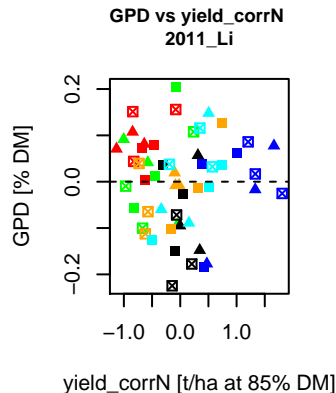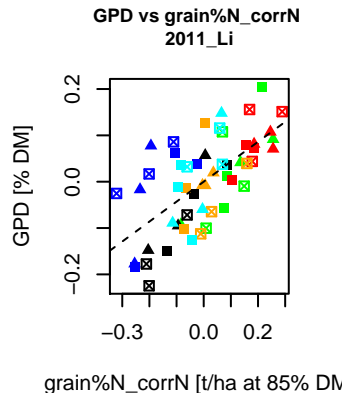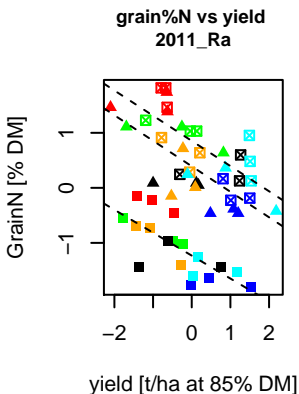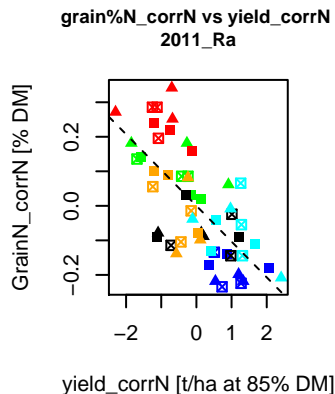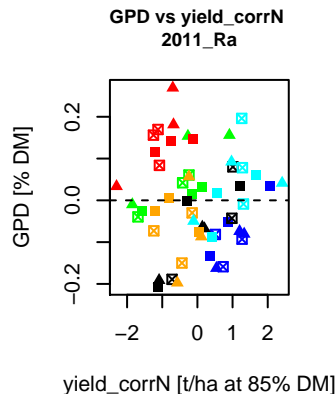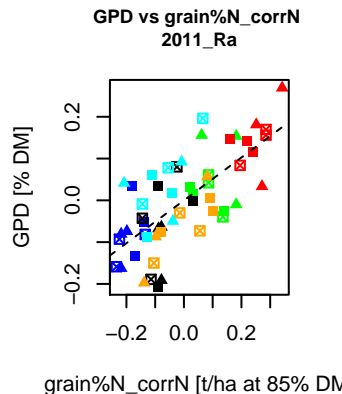

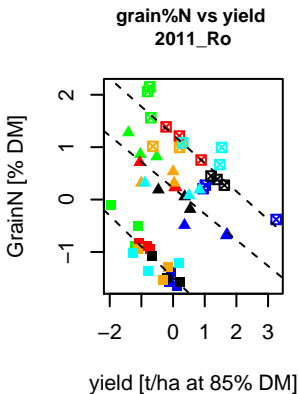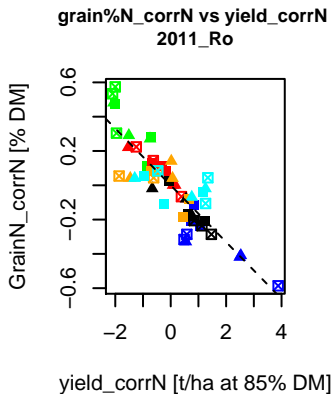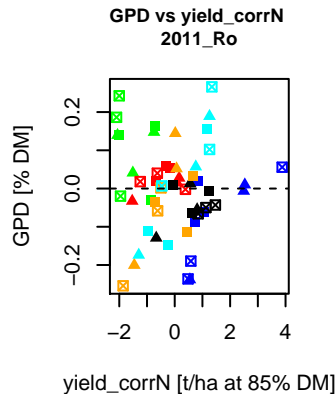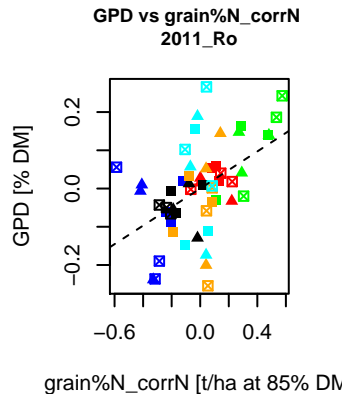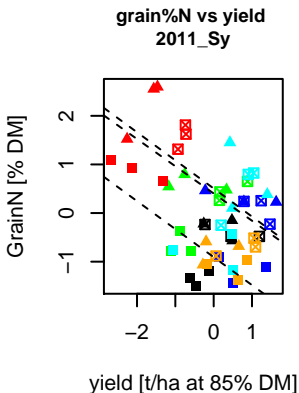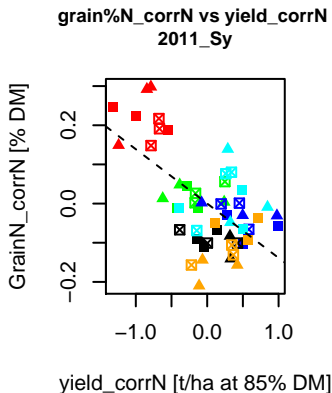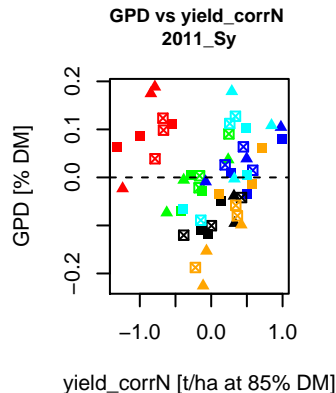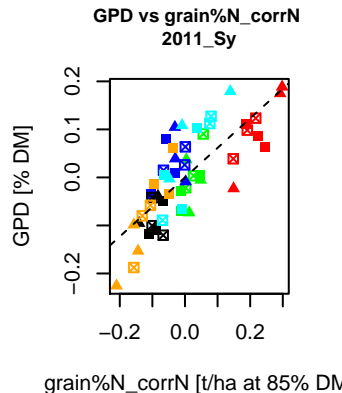

Supplement: Supplementary file 3 [file mmc3.pdf]
