# Supplementary material for: Genetic variation and heritability of grain protein deviation in European wheat genotypes
Source: Field Crops Res. 2020 Sep 15;255:107896. doi: 10.1016/j.fcr.2020.107896 (PMC7397848; doi:10.1016/j.fcr.2020.107896)
Supplement: Supplementary file 4 [file mmc4.pdf]

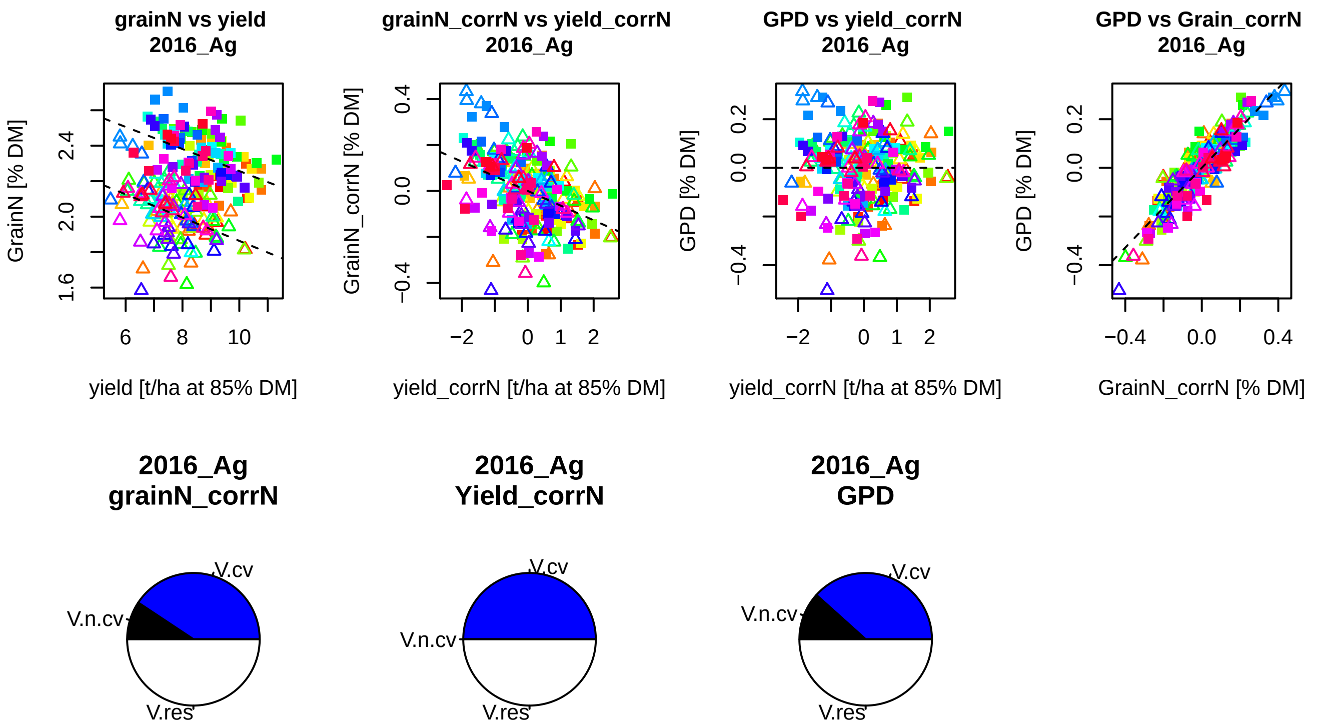

grainN vs yield
2016_Ag
GrainN [% DM]
1.6
2.0
2.4
6
8
10
yield [t/ha at 85% DM]
grainN_corrN vs yield_corrN
2016_Ag
GrainN_corrN [% DM]
−0.4
0.0
0.4
−2
0
1
2
yield_corrN [t/ha at 85% DM]
GPD vs yield_corrN
2016_Ag
GPD [% DM]
−0.4
0.0
0.2
−2
0
1
2
yield_corrN [t/ha at 85% DM]
GPD vs Grain_corrN
2016_Ag
GPD [% DM]
−0.4
0.0
0.2
−0.4
0.0
0.4
GrainN_corrN [% DM]
2016_Ag
grainN_corrN
V.cv
V.n.cv
V.res
2016_Ag
Yield_corrN
V.cv
V.n.cv
V.res
2016_Ag
GPD
V.cv
V.n.cv
V.res

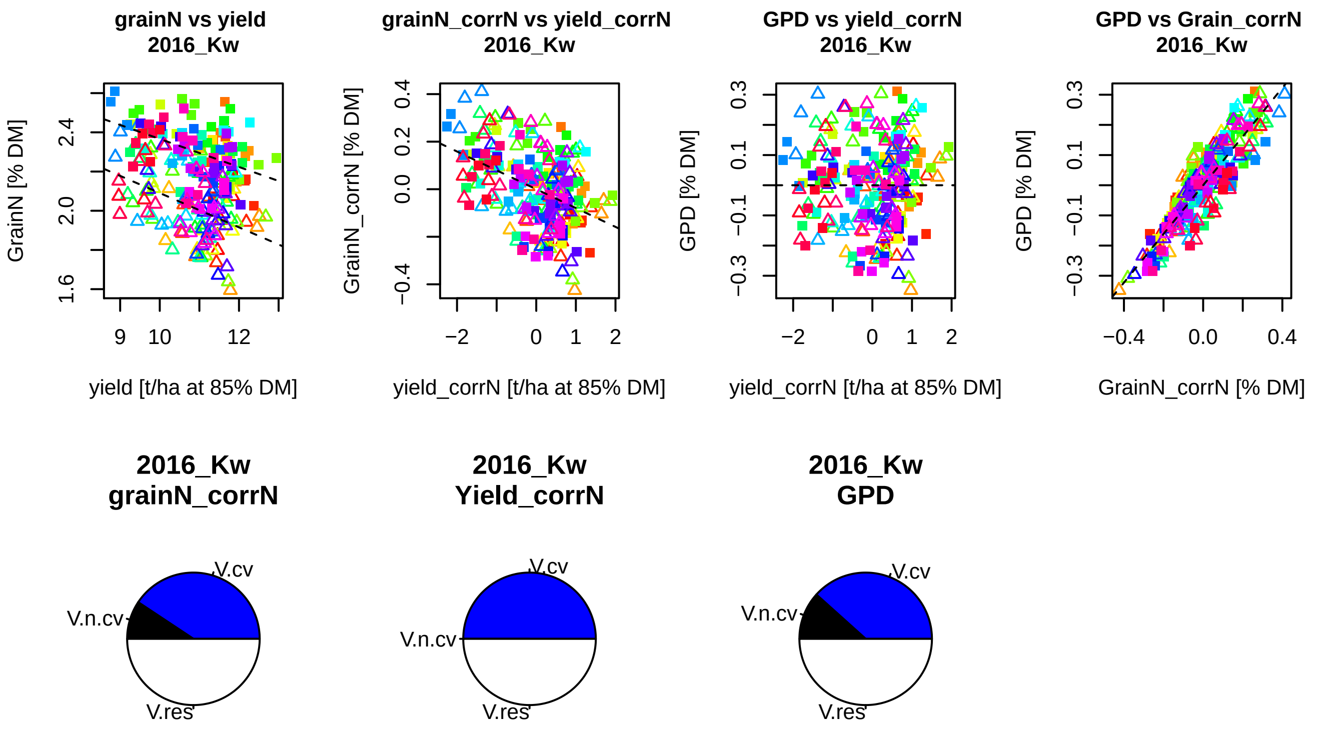
grainN vs yield
2016_Kw
grainN_corrN vs yield_corrN
2016_Kw
GPD vs yield_corrN
2016_Kw
GPD vs Grain_corrN
2016_Kw
GrainN [% DM]
1.6
2.0
2.4
9
10
12
yield [t/ha at 85% DM]
GrainN_corrN [% DM]
−0.4
0.0
0.2
0.4
−2
0
1
2
yield_corrN [t/ha at 85% DM]
GPD [% DM]
−0.3
−0.1
0.1
0.3
−2
0
1
2
yield_corrN [t/ha at 85% DM]
GPD [% DM]
−0.3
−0.1
0.1
0.3
−0.4
0.0
0.4
GrainN_corrN [% DM]
2016_Kw
grainN_corrN
V.cv
V.n.cv
V.res
2016_Kw
Yield_corrN
V.cv
V.n.cv
V.res
2016_Kw
GPD
V.cv
V.n.cv
V.res

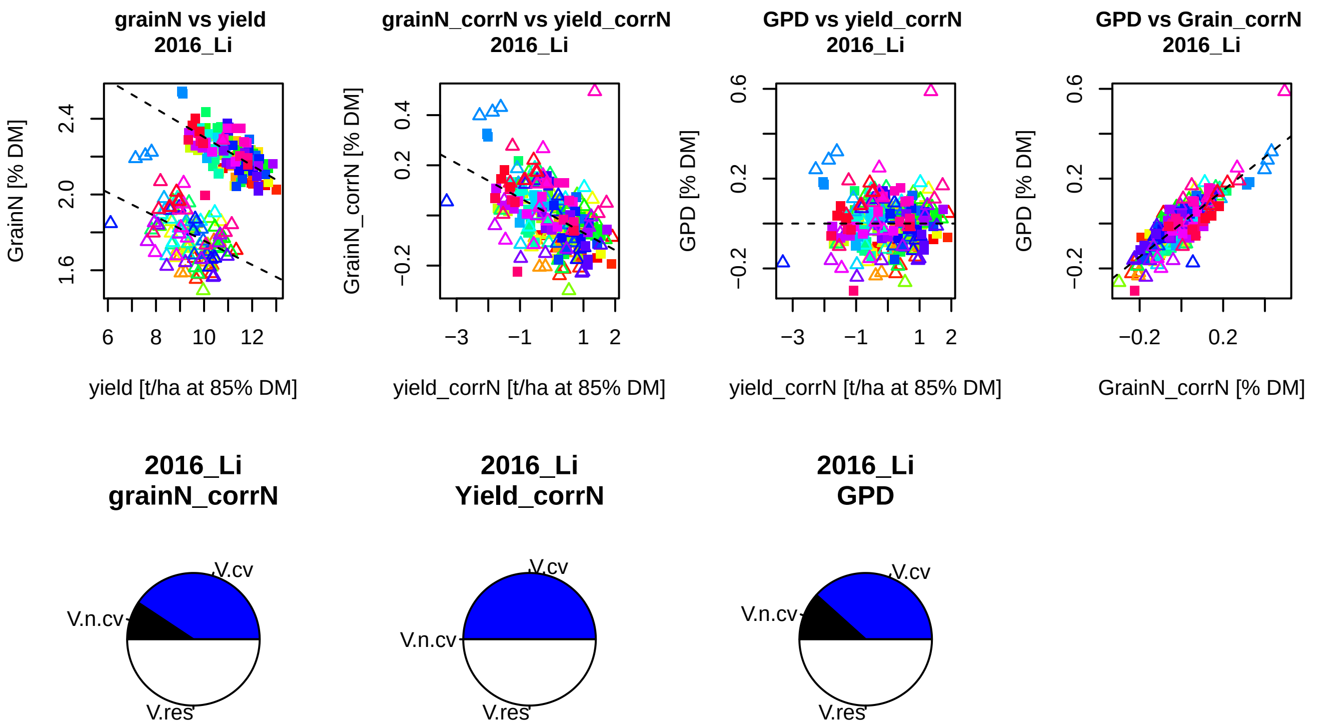

grainN vs yield
2016_Li
GrainN [% DM]
yield [t/ha at 85% DM]
grainN_corrN vs yield_corrN
2016_Li
GrainN_corrN [% DM]
yield_corrN [t/ha at 85% DM]
GPD vs yield_corrN
2016_Li
GPD [% DM]
yield_corrN [t/ha at 85% DM]
GPD vs Grain_corrN
2016_Li
GPD [% DM]
GrainN_corrN [% DM]
2016_Li
grainN_corrN
V.cv
V.n.cv
V.res
2016_Li
Yield_corrN
V.cv
V.n.cv
V.res
2016_Li
GPD
V.cv
V.n.cv
V.res

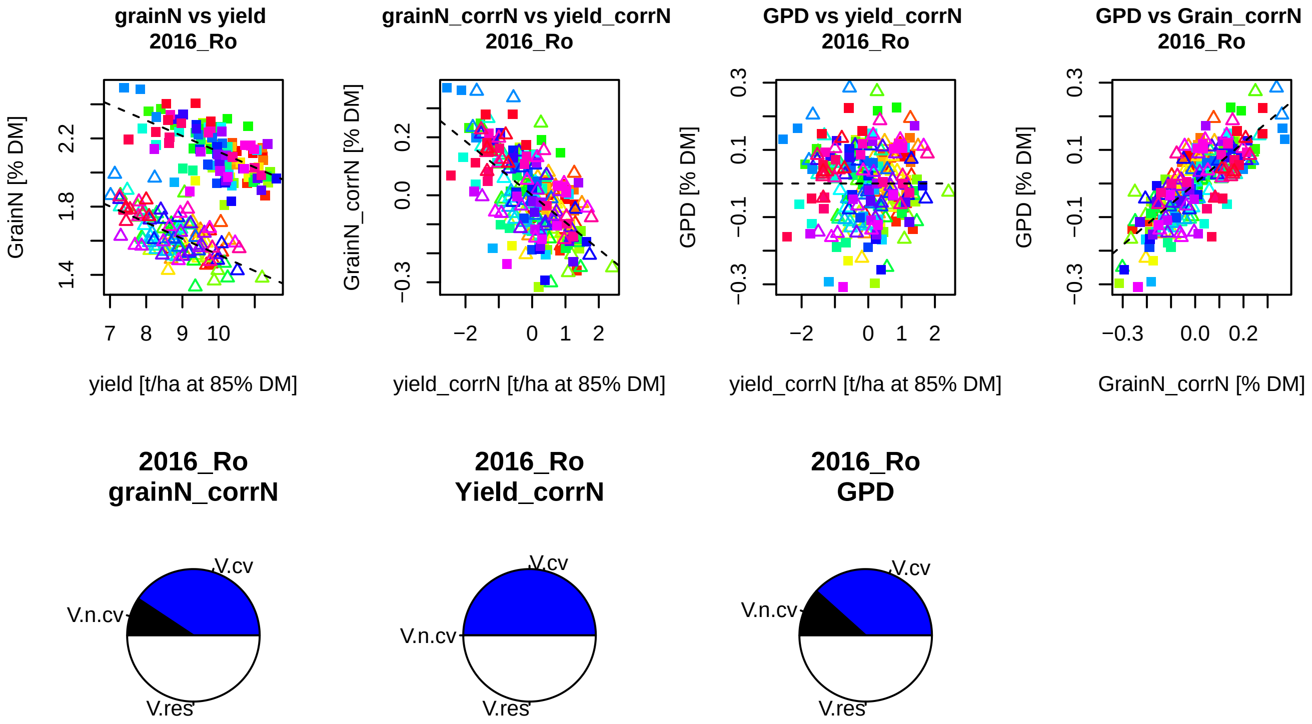
grainN vs yield
2016_Ro
GrainN [% DM]
1.4
1.8
2.2
7
8
9
10
yield [t/ha at 85% DM]
grainN_corrN vs yield_corrN
2016_Ro
GrainN_corrN [% DM]
−0.3
0.0
0.2
−2
0
1
2
yield_corrN [t/ha at 85% DM]
GPD vs yield_corrN
2016_Ro
GPD [% DM]
−0.3
−0.1
0.1
0.3
−2
0
1
2
yield_corrN [t/ha at 85% DM]
GPD vs Grain_corrN
2016_Ro
GPD [% DM]
−0.3
−0.1
0.1
0.3
−0.3
0.0
0.2
GrainN_corrN [% DM]
2016_Ro
grainN_corrN
V.cv
V.n.cv
V.res
2016_Ro
Yield_corrN
V.cv
V.n.cv
V.res
2016_Ro
GPD
V.cv
V.n.cv
V.res

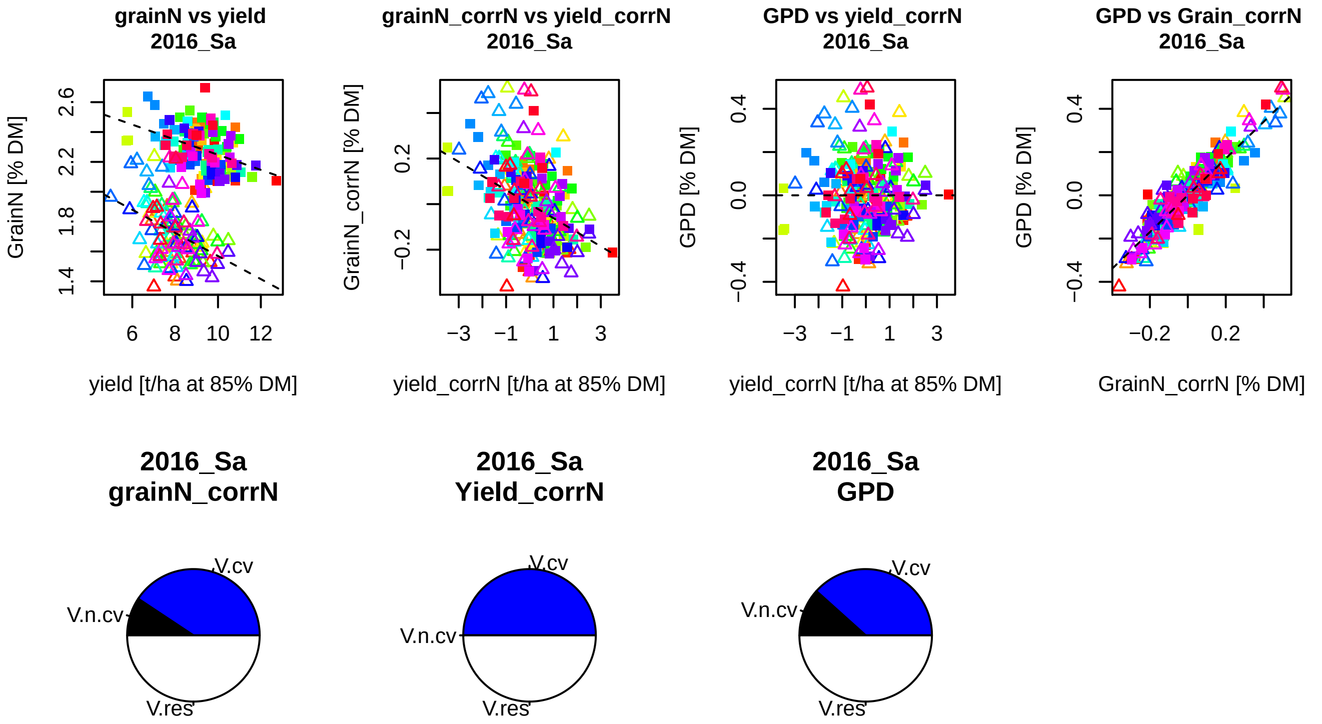
grainN vs yield
2016_Sa
GrainN [% DM]
yield [t/ha at 85% DM]
grainN_corrN vs yield_corrN
2016_Sa
GrainN_corrN [% DM]
yield_corrN [t/ha at 85% DM]
GPD vs yield_corrN
2016_Sa
GPD [% DM]
yield_corrN [t/ha at 85% DM]
GPD vs Grain_corrN
2016_Sa
GPD [% DM]
GrainN_corrN [% DM]
2016_Sa
grainN_corrN
V.cv
V.n.cv
V.res
2016_Sa
Yield_corrN
V.cv
V.n.cv
V.res
2016_Sa
GPD
V.cv
V.n.cv
V.res

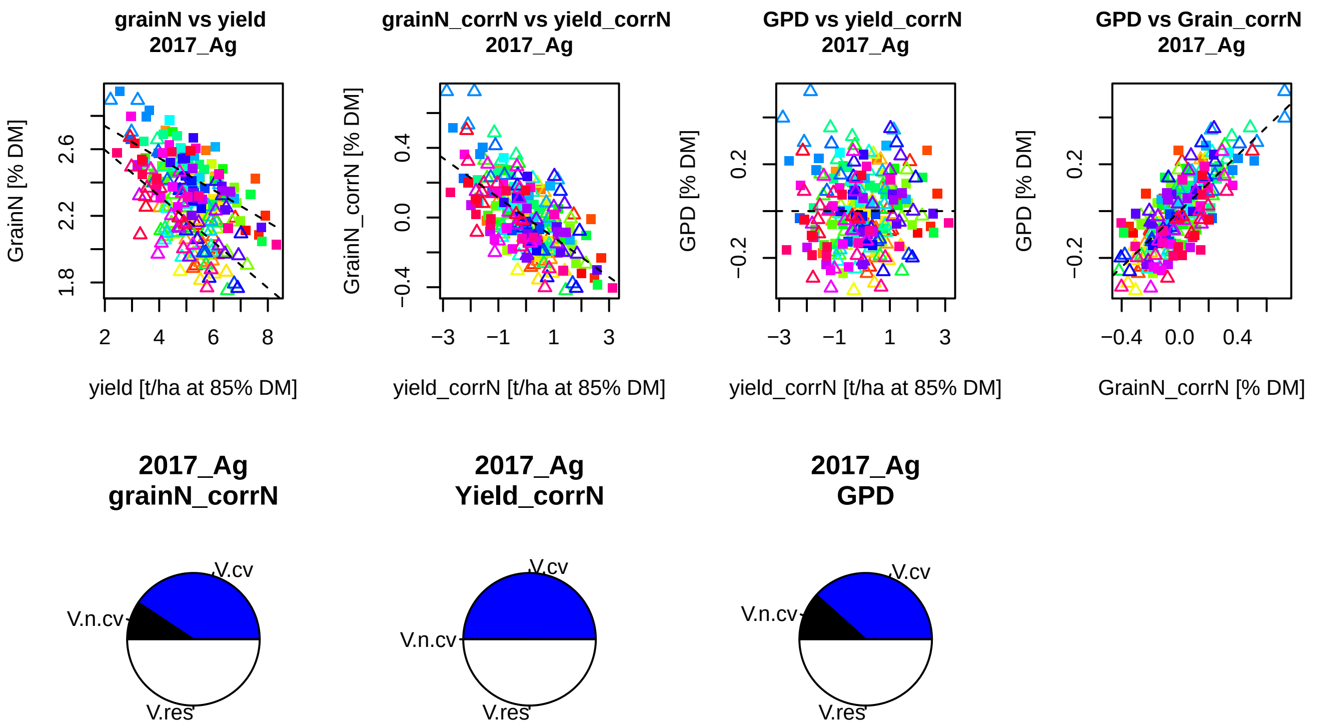

grainN vs yield
2017_Ag
GrainN [% DM]
yield [t/ha at 85% DM]
grainN_corrN vs yield_corrN
2017_Ag
GrainN_corrN [% DM]
yield_corrN [t/ha at 85% DM]
GPD vs yield_corrN
2017_Ag
GPD [% DM]
yield_corrN [t/ha at 85% DM]
GPD vs Grain_corrN
2017_Ag
GPD [% DM]
GrainN_corrN [% DM]
2017_Ag
grainN_corrN
V.cv
V.n.cv
V.res
2017_Ag
Yield_corrN
V.cv
V.n.cv
V.res
2017_Ag
GPD
V.cv
V.n.cv
V.res

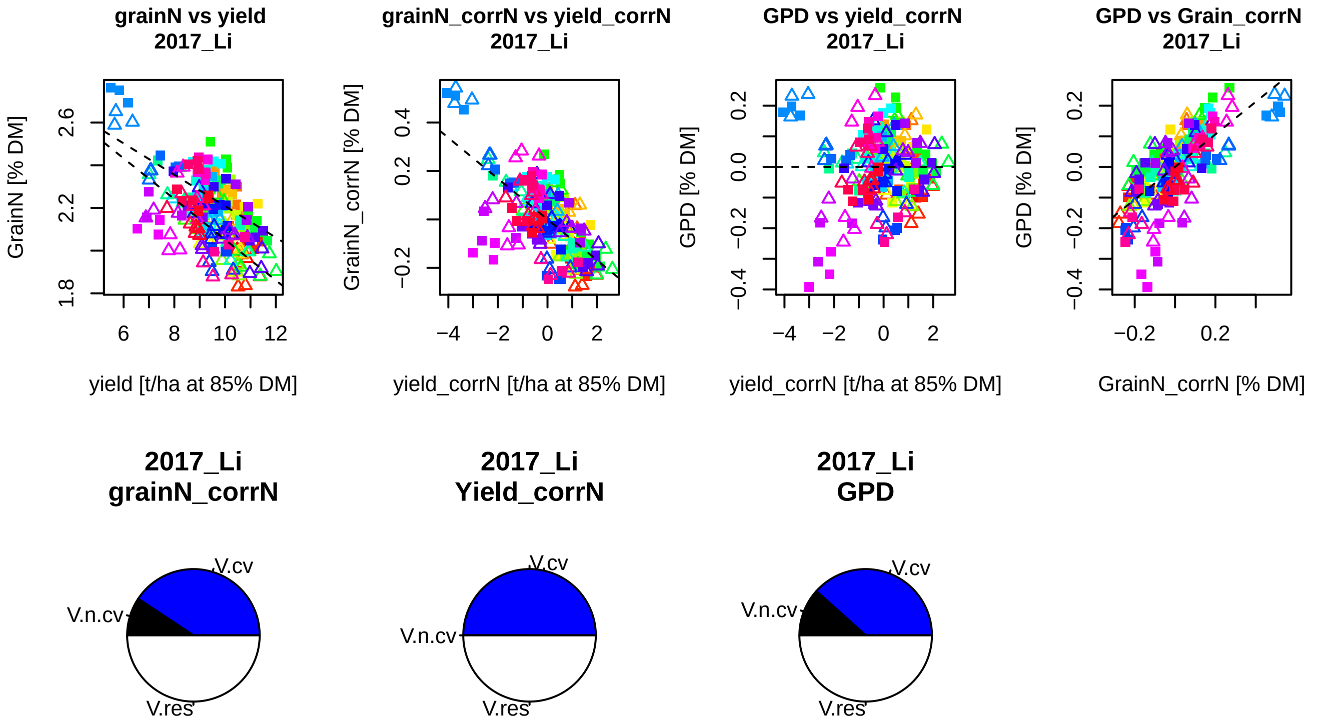

grainN vs yield
2017_Li
GrainN [% DM]
yield [t/ha at 85% DM]
1.8
2.2
2.6
6
8
10
12
grainN_corrN vs yield_corrN
2017_Li
GrainN_corrN [% DM]
yield_corrN [t/ha at 85% DM]
−0.2
0.2
0.4
−4
−2
0
2
GPD vs yield_corrN
2017_Li
GPD [% DM]
yield_corrN [t/ha at 85% DM]
−0.4
−0.2
0.0
0.2
−4
−2
0
2
GPD vs Grain_corrN
2017_Li
GPD [% DM]
GrainN_corrN [% DM]
−0.4
−0.2
0.0
0.2
−0.2
0.2
2017_Li
grainN_corrN
V.cv
V.n.cv
V.res
2017_Li
Yield_corrN
V.cv
V.n.cv
V.res
2017_Li
GPD
V.cv
V.n.cv
V.res

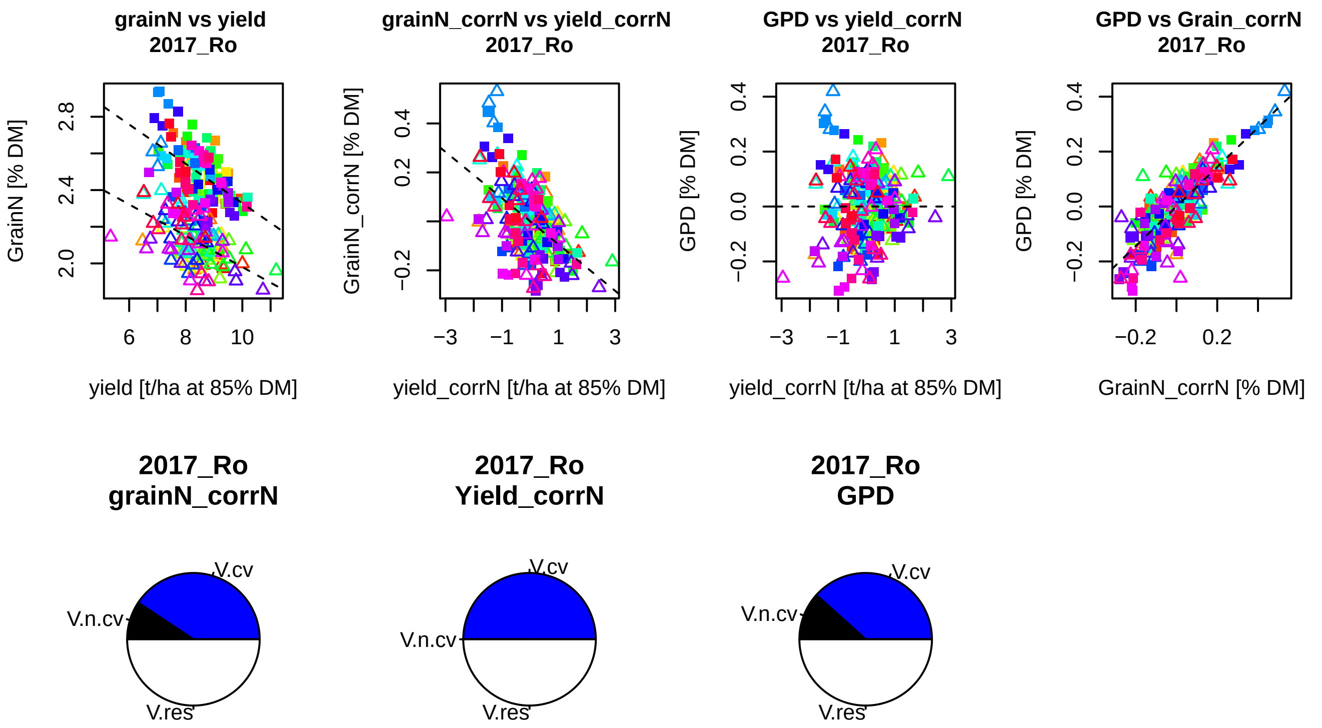

grainN vs yield
2017_Ro
GrainN [% DM]
yield [t/ha at 85% DM]
grainN_corrN vs yield_corrN
2017_Ro
GrainN_corrN [% DM]
yield_corrN [t/ha at 85% DM]
GPD vs yield_corrN
2017_Ro
GPD [% DM]
yield_corrN [t/ha at 85% DM]
GPD vs Grain_corrN
2017_Ro
GPD [% DM]
GrainN_corrN [% DM]
2017_Ro
grainN_corrN
V.cv
V.n.cv
V.res
2017_Ro
Yield_corrN
V.cv
V.n.cv
V.res
2017_Ro
GPD
V.cv
V.n.cv
V.res

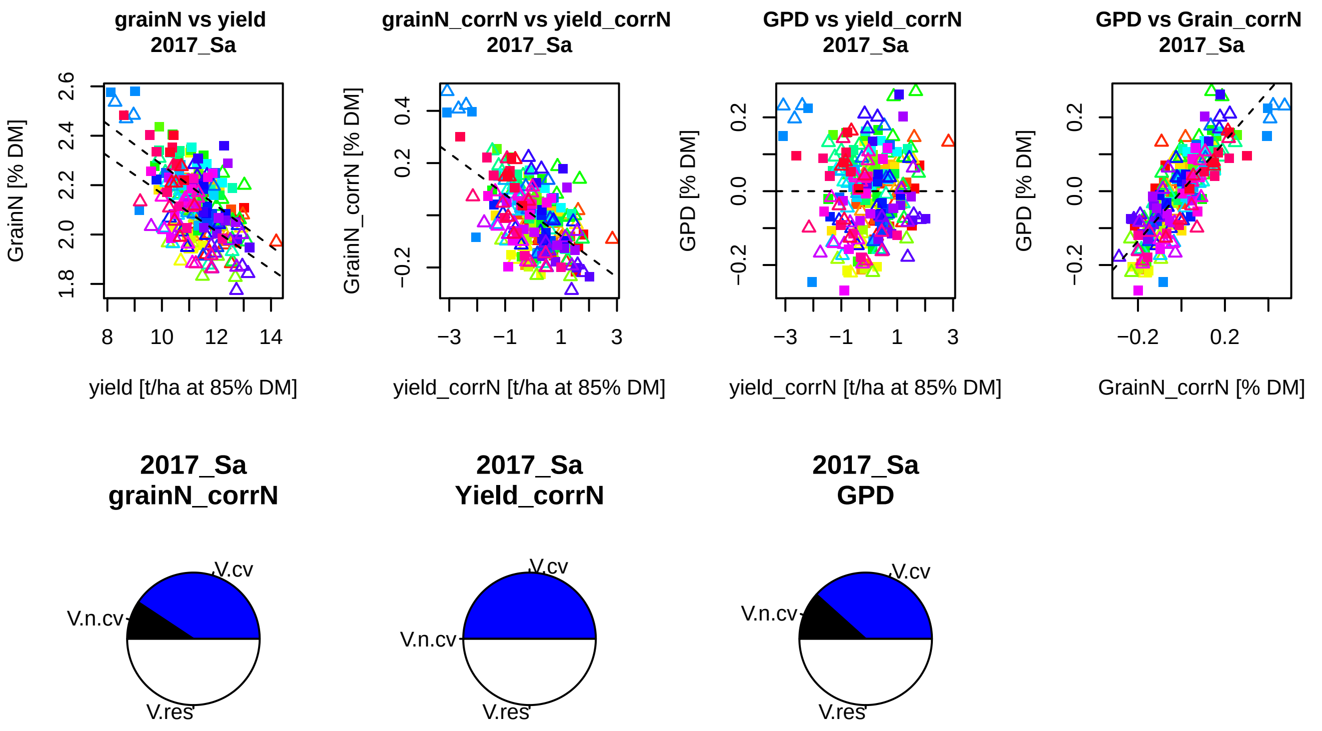
grainN vs yield
2017_Sa
GrainN [% DM]
1.8
2.0
2.2
2.4
2.6
8
10
12
14
yield [t/ha at 85% DM]
grainN_corrN vs yield_corrN
2017_Sa
GrainN_corrN [% DM]
−0.2
0.2
0.4
−3
−1
1
3
yield_corrN [t/ha at 85% DM]
GPD vs yield_corrN
2017_Sa
GPD [% DM]
−0.2
0.0
0.2
−3
−1
1
3
yield_corrN [t/ha at 85% DM]
GPD vs Grain_corrN
2017_Sa
GPD [% DM]
−0.2
0.0
0.2
−0.2
0.2
GrainN_corrN [% DM]
2017_Sa
grainN_corrN
V.cv
V.n.cv
V.res
2017_Sa
Yield_corrN
V.cv
V.n.cv
V.res
2017_Sa
GPD
V.cv
V.n.cv
V.res

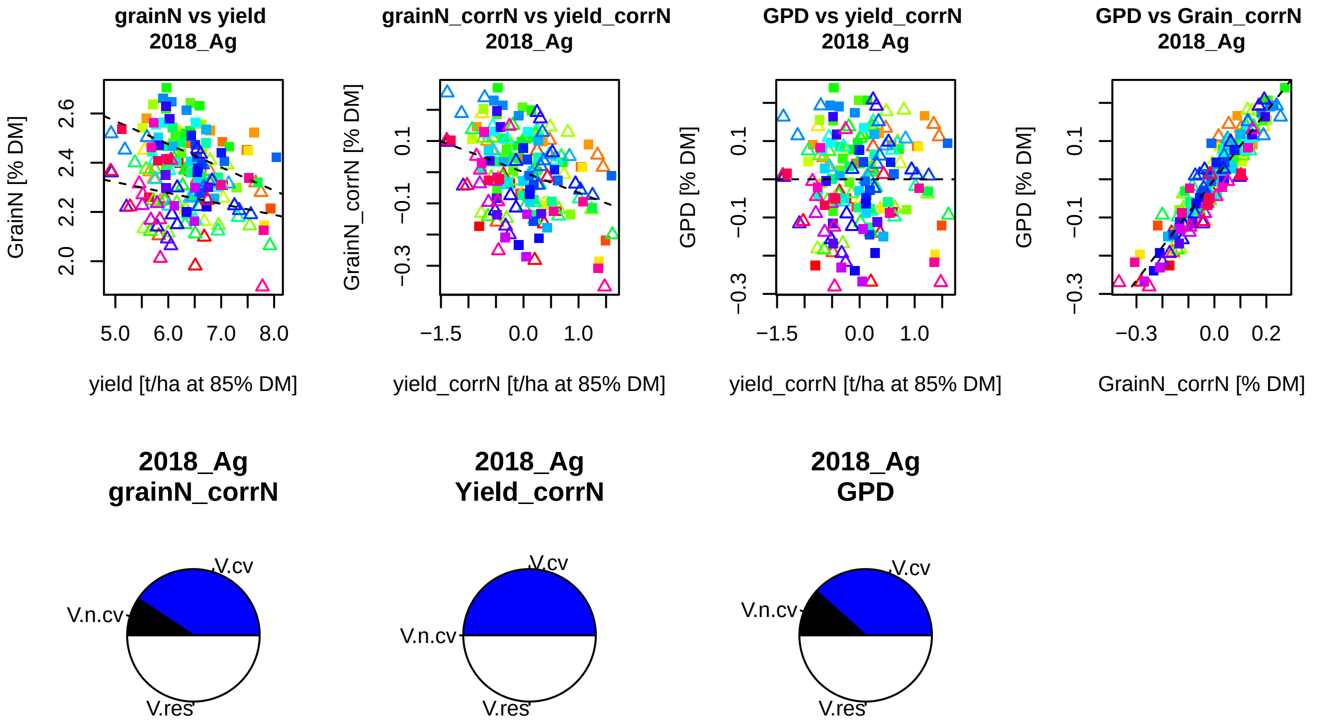
grainN vs yield
2018_Ag
GrainN [% DM]
yield [t/ha at 85% DM]
grainN_corrN vs yield_corrN
2018_Ag
GrainN_corrN [% DM]
yield_corrN [t/ha at 85% DM]
GPD vs yield_corrN
2018_Ag
GPD [% DM]
yield_corrN [t/ha at 85% DM]
GPD vs Grain_corrN
2018_Ag
GPD [% DM]
GrainN_corrN [% DM]
2018_Ag
grainN_corrN
V.cv
V.n.cv
V.res
2018_Ag
Yield_corrN
V.cv
V.n.cv
V.res
2018_Ag
GPD
V.cv
V.n.cv
V.res

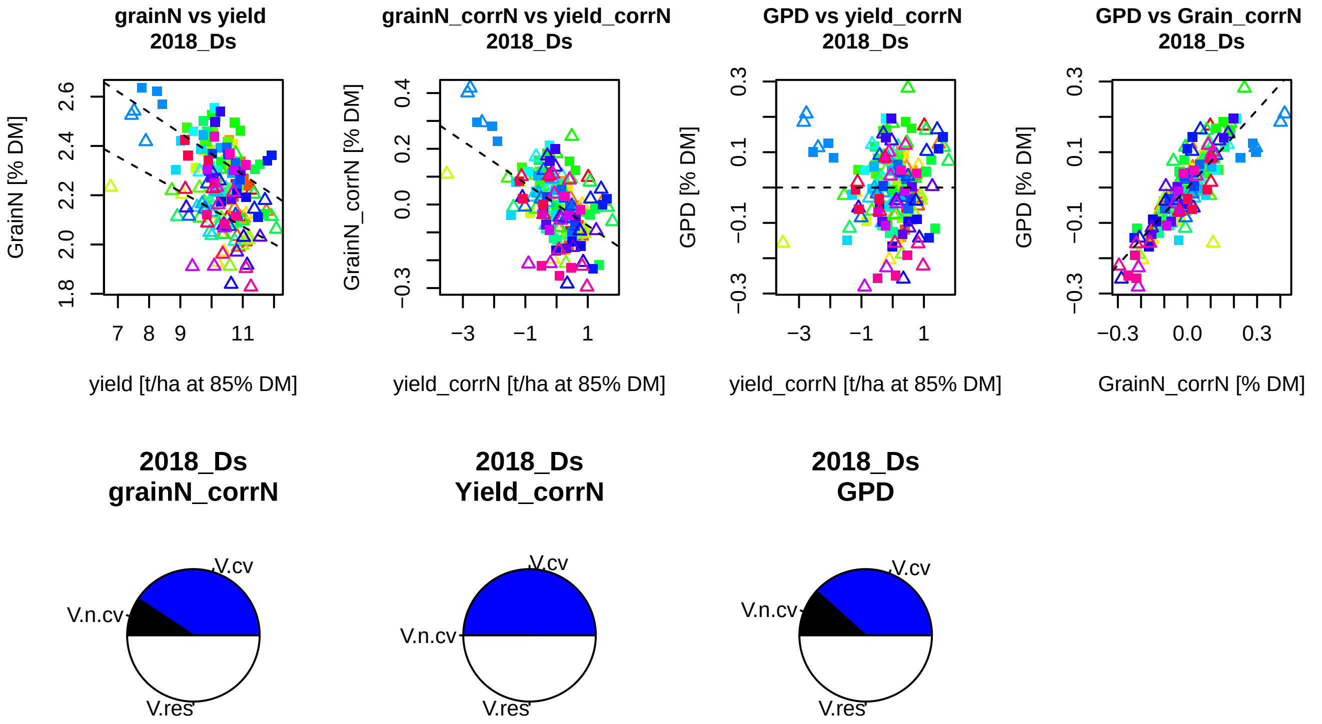
grainN vs yield
2018_Ds
GrainN [% DM]
yield [t/ha at 85% DM]
grainN_corrN vs yield_corrN
2018_Ds
GrainN_corrN [% DM]
yield_corrN [t/ha at 85% DM]
GPD vs yield_corrN
2018_Ds
GPD [% DM]
yield_corrN [t/ha at 85% DM]
GPD vs Grain_corrN
2018_Ds
GPD [% DM]
GrainN_corrN [% DM]
2018_Ds
grainN_corrN
V.cv
V.n.cv
V.res
2018_Ds
Yield_corrN
V.cv
V.n.cv
V.res
2018_Ds
GPD
V.cv
V.n.cv
V.res

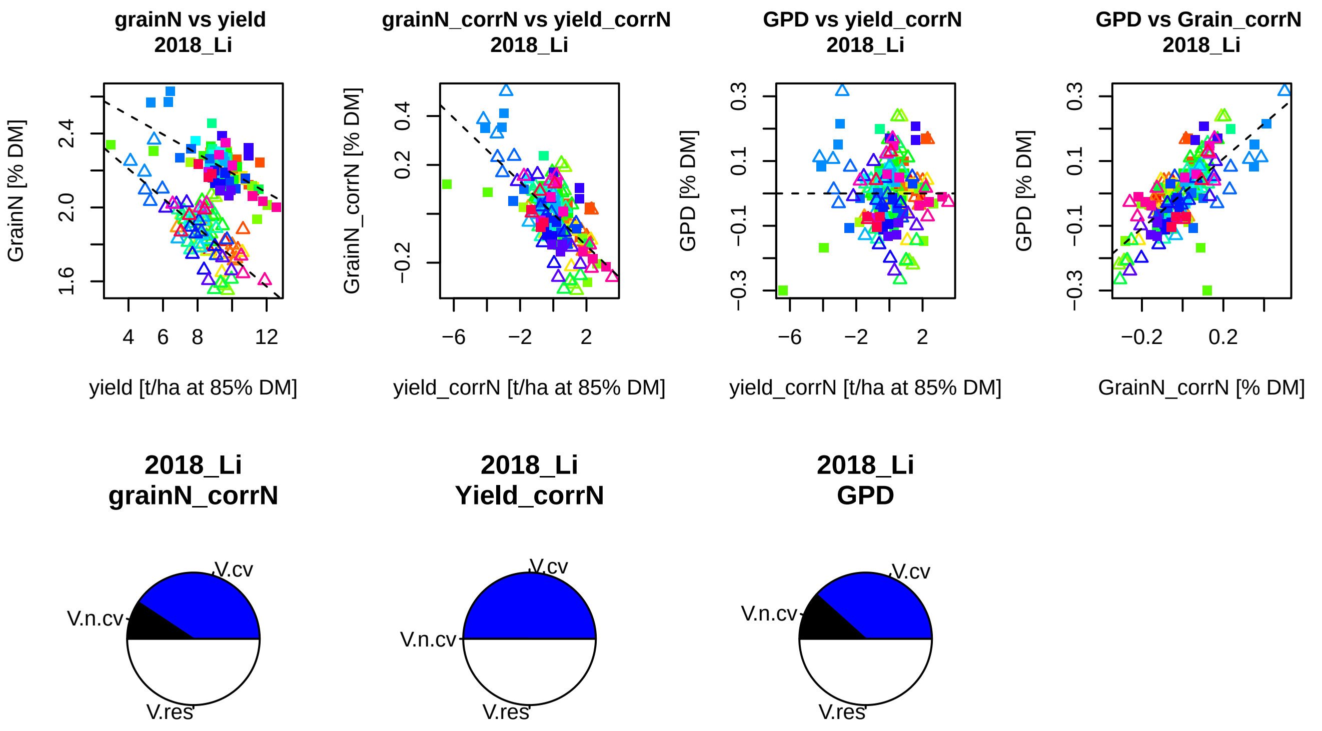
grainN vs yield
2018_Li
GrainN [% DM]
yield [t/ha at 85% DM]
1.6
2.0
2.4
4
6
8
12
grainN_corrN vs yield_corrN
2018_Li
GrainN_corrN [% DM]
yield_corrN [t/ha at 85% DM]
−0.2
0.2
0.4
−6
−2
2
GPD vs yield_corrN
2018_Li
GPD [% DM]
yield_corrN [t/ha at 85% DM]
−0.3
−0.1
0.1
0.3
−6
−2
2
GPD vs Grain_corrN
2018_Li
GPD [% DM]
GrainN_corrN [% DM]
−0.3
−0.1
0.1
0.3
−0.2
0.2
2018_Li
grainN_corrN
V.cv
V.n.cv
V.res
2018_Li
Yield_corrN
V.cv
V.n.cv
V.res
2018_Li
GPD
V.cv
V.n.cv
V.res

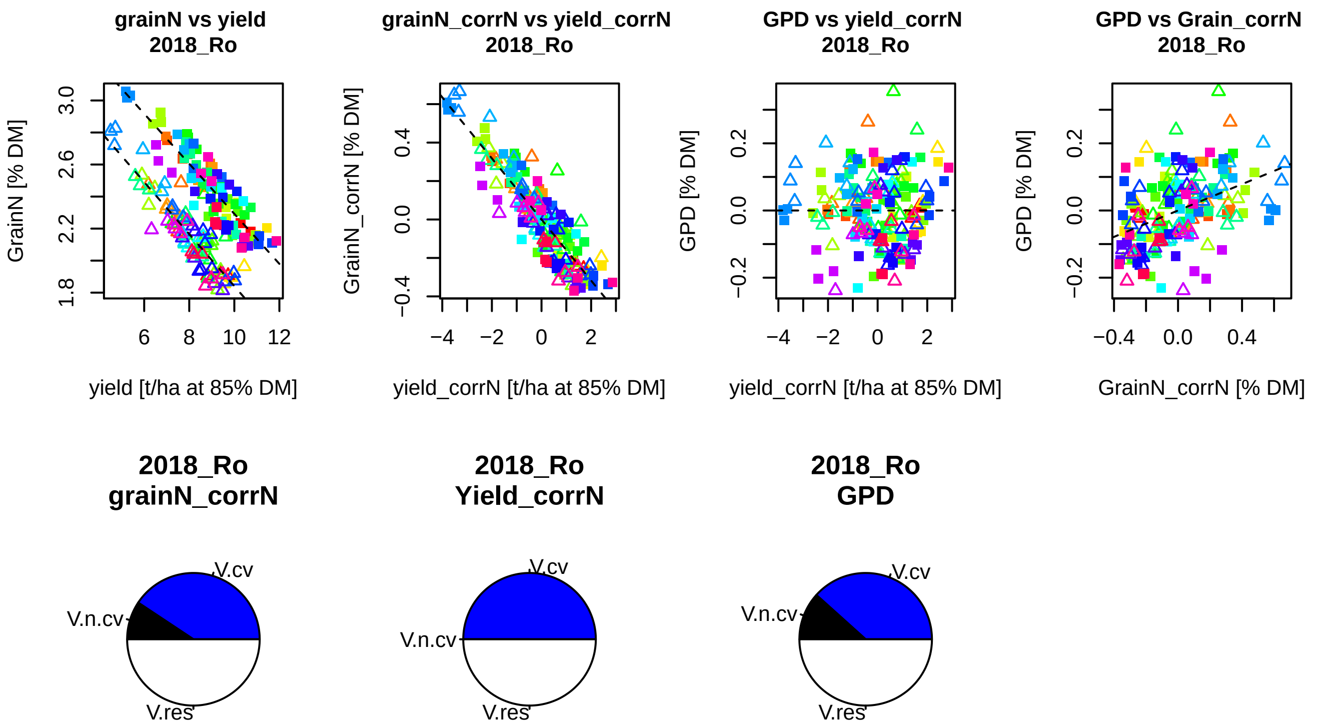
grainN vs yield
2018_Ro
GrainN [% DM]
3.0
2.6
2.2
1.8
6
8
10
12
yield [t/ha at 85% DM]
grainN_corrN vs yield_corrN
2018_Ro
GrainN_corrN [% DM]
0.4
0.0
−0.4
−4
−2
0
2
yield_corrN [t/ha at 85% DM]
GPD vs yield_corrN
2018_Ro
GPD [% DM]
0.2
0.0
−0.2
−4
−2
0
2
yield_corrN [t/ha at 85% DM]
GPD vs Grain_corrN
2018_Ro
GPD [% DM]
0.2
0.0
−0.2
−0.4
0.0
0.4
GrainN_corrN [% DM]
2018_Ro
grainN_corrN
V.cv
V.n.cv
V.res
2018_Ro
Yield_corrN
V.cv
V.n.cv
V.res
2018_Ro
GPD
V.cv
V.n.cv
V.res

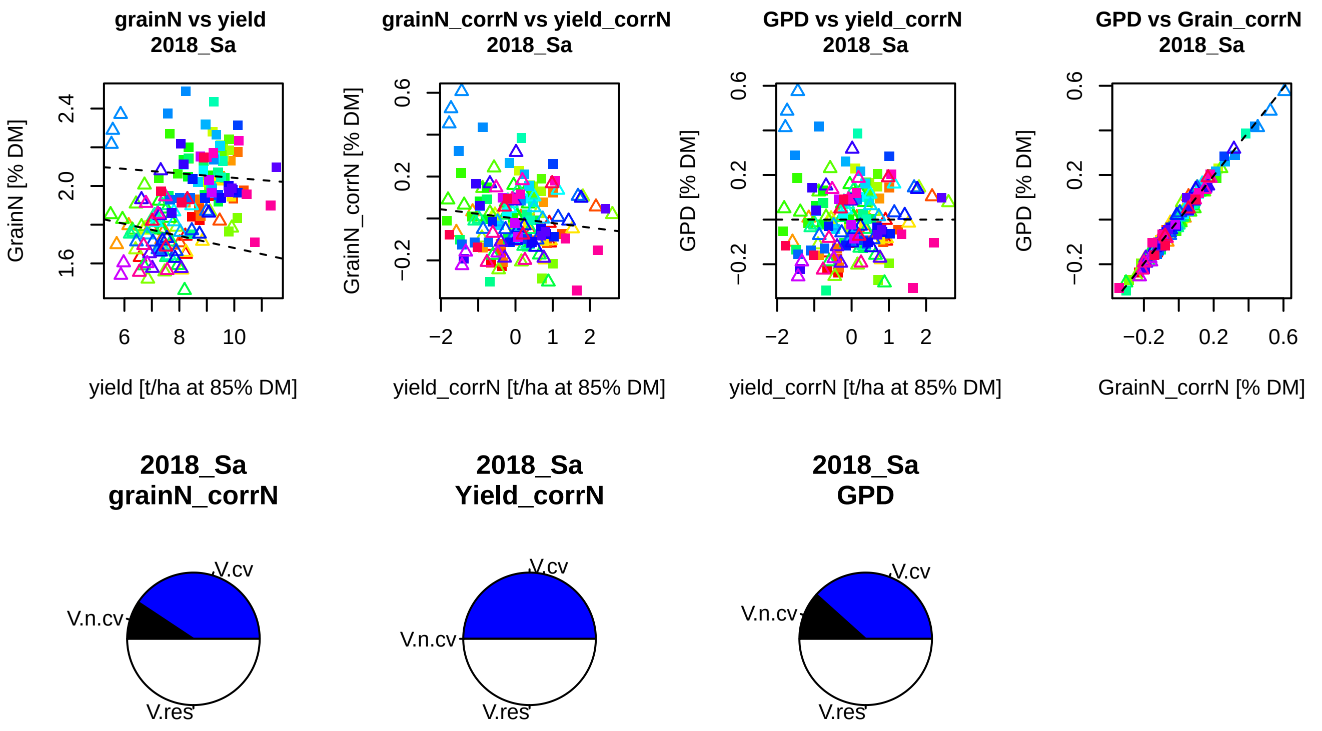
grainN vs yield
2018_Sa
GrainN [% DM]
yield [t/ha at 85% DM]
1.6
2.0
2.4
6
8
10
grainN_corrN vs yield_corrN
2018_Sa
GrainN_corrN [% DM]
yield_corrN [t/ha at 85% DM]
−0.2
0.2
0.6
−2
0
1
2
GPD vs yield_corrN
2018_Sa
GPD [% DM]
yield_corrN [t/ha at 85% DM]
−0.2
0.2
0.6
−2
0
1
2
GPD vs Grain_corrN
2018_Sa
GPD [% DM]
GrainN_corrN [% DM]
−0.2
0.2
0.6
−0.2
0.2
0.6
2018_Sa
grainN_corrN
V.cv
V.n.cv
V.res
2018_Sa
Yield_corrN
V.cv
V.n.cv
V.res
2018_Sa
GPD
V.cv
V.n.cv
V.res
