# Supplementary material for: Genetic variation and heritability of grain protein deviation in European wheat genotypes
Source: Field Crops Res. 2020 Sep 15;255:107896. doi: 10.1016/j.fcr.2020.107896 (PMC7397848; doi:10.1016/j.fcr.2020.107896)
Supplement: Supplementary file 5 [file mmc5.docx]

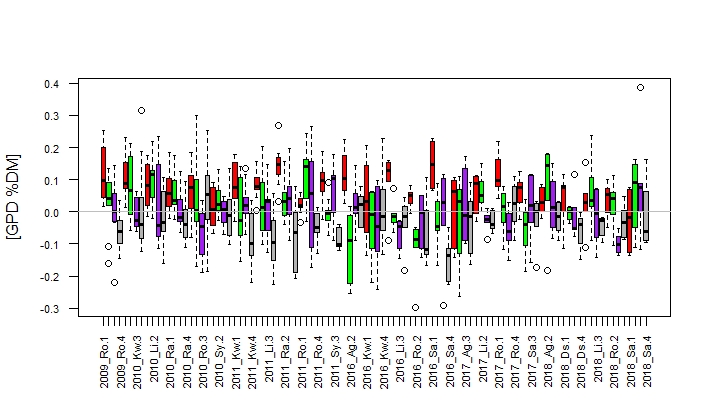


Figure. S3. Boxplot of GPD for the four cultivars grown in all years: Hereward (red), Cordiale (green), Xi19 (purpe), and Malacca (grey).

Each box shows variation across the sites and replicates within each year and cultivar. The sites are indicated by their two first letters: Ro (Rothamsted), Kw (KWS), Li (Limagrain), Ra (RAGT), Sy (Syngenta), Ds (DSV), Sa (Saaten Union) and Ag (Agrii) The axis label represent every third box which is sufficient for the presentation of the location. The final numbers are the cultivars, which are also colour coded (see above).
