# Supplementary material for: Genetic variation and heritability of grain protein deviation in European wheat genotypes
Source: Field Crops Res. 2020 Sep 15;255:107896. doi: 10.1016/j.fcr.2020.107896 (PMC7397848; doi:10.1016/j.fcr.2020.107896)
Supplement: Supplementary file 6 [file mmc6.docx]

Supplementary Table S1. Cultivars selected for the field trials in 2015-2016, 2016-2017 and 2017-2018. Those not grown in 2017-2018 are shown in red. The NABIM groups are used in the UK to classify wheat for different end used.

| **Number** | **Type** | **Cultivar** | **Number** | **Type** |  |
| --- | --- | --- | --- | --- | --- |
| 1 | NABIN group 4 (feed)  winter type | JB Diego | 21 | Older UK | Cadenza |
| 2 |  | Dickens | 22 |  | Malacca |
| 3 | UK NABIN group 1 (best quality breadmaking)  winter type | Skyfall | 23 |  | Shamrock |
| 4 |  | Crusoe | 24 | Hungarian high protein wheats | Mv Karisma |
| 5 |  | Gallant | 25 |  | Mv Lucilla |
| 6 |  | Solstice | 26 | German | Memory |
| 7 |  | KWS Trinity | 27 |  | Potenzial |
| 8 | UK NABIM group 2 (breadmaking)  winter type | Einstein | 28 |  | Rumor |
| 9 |  | KWS Cashel | 29 |  | Nelson |
| 10 |  | Cordiale | 30 | French hybrid wheats | Hybery SU |
| 11 |  | KWS Lili | 31 |  | Hystar |
| 12 | UK Spring type | Mulika | 32 | French | Tobak |
| 13 |  | Paragon | 33 | German | Apache |
| 14 |  | Granary | 34 |  | Arlequin |
| 15 |  | KWS Willow | 35 |  | Premio |
| 16 | Older UK | KWS Siskin | 36 | Danish | Genius |
| 17 |  | Hereward | 37 |  | Dacanto |
| 18 |  | Soissons | 38 | Genetic lines in UK spring wheat Paragon | Paragon 1BL/1RS |
| 19 |  | Xi19 | 39 |  | Paragon Stay Green |
| 20 |  | Avalon | 40 |  | Paragon Rht2 |
