# Supplementary material for: Genetic variation and heritability of grain protein deviation in European wheat genotypes
Source: Field Crops Res. 2020 Sep 15;255:107896. doi: 10.1016/j.fcr.2020.107896 (PMC7397848; doi:10.1016/j.fcr.2020.107896)
Supplement: Supplementary file 7 [file mmc7.docx]

**Supplementary Table S2**. Pairwise comparisons of means of the four cultivars present in all year of growth with Tukey’s test (95% confidence interval)

|  | **Yield** | | **GrainN** | | **Yield_corrN** |  | **GrainN_corrN** | | **GPD** | |
| --- | --- | --- | --- | --- | --- | --- | --- | --- | --- | --- |
| **He** | 8.24 | C | 2.26 | A | -0.64 | D | 0.13 | A | 0.070 | A |
| **Co** | 8.85 | A,B | 2.17 | B | -0.03 | B | 0.04 | B | 0.022 | B |
| **Xi** | 9.14 | A | 2.10 | B | 0.26 | A | -0.03 | C | 0.000 | B |
| **Ma** | 8.63 | B,C | 2.11 | B | -0.26 | C | -0.02 | C | -0.037 | C |
